# Signal-integrated control of immune and reparative programs establishes blood-derived fibrocytes reprogrammable toward induced pluripotency

**DOI:** 10.1242/bio.062486

**Published:** 2026-09-29

**Authors:** Hannah Sylvester, Prasanthi P. Koganti, Shailesh Gurung, Viju V. Pillai, Vimal Selvaraj

**Affiliations:** ^1^ Department of Animal Science, College of Agriculture and Life Sciences, Cornell University, Ithaca, NY 14853, USA; ^2^ Department of Comparative Pathobiology, College of Veterinary Medicine, Purdue University, West Lafayette, IN 47907, USA

**Keywords:** Stem cells, Conservation, Genetic preservation, Reproduction, Biotechnology

## Abstract

Fibrocytes represent a distinct somatic cell type derived from peripheral blood leukocytes, first described as spindle-shaped adherent cells with dual hematopoietic and mesenchymal features. Although fibrocytes were identified in early descriptive studies across several mammalian systems, most of this work predated modern molecular approaches, and the cells remain incompletely defined at the molecular level and have not previously been derived or characterized in cattle. Seeking somatic cells that could be collected aseptically and reproducibly under field conditions for reprogramming to pluripotency, we recognized fibrocytes as a practical and previously unexplored candidate population. Here, we establish a reproducible method for fibrocyte derivation and expansion from adult bovine blood and define their molecular identity using transcriptomic and network analyses. Principal component and differential expression analyses revealed extensive immune, inflammatory, metabolic, and stress-responsive pathways that distinguished fibrocytes from fibroblasts. Upstream regulator analysis identified a fibrocyte-restricted transcriptional network governed by SPI1, IRF5/IRF7, NFKBIZ, PRDM1, CIITA, and MAFB, supporting a monocyte-derived origin and indicating some retention of hematopoietic lineage memory despite acquisition of mesenchymal features. Optimized fibrocyte medium (dexamethasone, ascorbate, platelet-derived growth factor-BB, epidermal growth factor, A83-01, CHIR99021) supported stable proliferation and selectively enhanced cytoskeletal and matrix-constructive programs while attenuating inflammatory tone. As a proof of principle, fibrocytes reprogrammed with polycistronic OCT4-SOX2-KLF4-cMYC and SV40 large T antigen generated induced pluripotent stem cell colonies exhibiting defining molecular and morphological features of pluripotency. These findings establish fibrocytes as a field-adaptable, stably expandable, and reprogrammable somatic cell type with practical applications in induced pluripotent stem cell generation, genetic preservation, and reproductive biotechnology.

## Teaser

**Summary:** Culture and characterization of bovine blood-derived fibrocytes reveal a promising, accessible somatic cell source for induced pluripotent stem cell generation.

## INTRODUCTION

The concept of circulating cells in blood contributing to tissue repair dates back over a century. In 1922, it was demonstrated that cells assuming the appearance of fibroblasts could be cultured from blood of adult chickens (Carrel and Ebeling, 1922). At the time, the origin of these cells remained speculative, with postulates suggesting a transformation of circulating monocytes, or a fibroblastic cell type that exists in small numbers in peripheral blood. In 1927, it was demonstrated that fibroblast-like cells with indistinguishable morphology from connective tissue fibroblasts could be cultured from guinea pig blood (Maximow, 1927). These observations were later extended to human peripheral blood in 1958, confirming that fibroblast-like cells could be propagated from cells in adult human circulation (Paul, 1958). Supporting these findings, radiolabeled tracer studies linked circulating leukocytes to new connective tissue formation during wound repair (MacDonald, 1959). Further validation came from experiments in which human mononuclear leukocytes, cultured in diffusion chambers *in vivo*, differentiated into ‘polyblasts’ before assuming the morphological characteristics of collagen-producing fibroblast-like cells (Hjortdal and Rasmussen, 1969; Petrakis et al., 1961). These results were consistently reproduced across different mammalian species using various methodologies (Luria et al., 1971; Stirling and Kakkar, 1969). While the precise cellular origin of these fibroblast-like cells remained uncertain, the consensus emerged that a subset, termed ‘fibrocytes’, originated from circulating mononuclear cells and contributed to wound repair (Grillo, 1964).

The search for the precise identity of fibrocytes in peripheral blood remained an active area of investigation for several decades. Although the notion of fibrocytes as a *de novo* rare leukocyte subpopulation remains plausible (Bucala et al., 1994), the extended culture times required for their detection do not exclude the likely possibility of trans-differentiation in their derivation. Subsequent studies have demonstrated the transformation of monocytes into fibroblast-like fibrocytes, solidifying their identity as neo-fibroblasts (Labat et al., 1994). Additionally, fibrocytes were found to retain epigenetic markers indicative of their hematopoietic lineage. They continued to express overlapping surface markers such as CD11b, CD13, CD34, and CD45 (Bucala et al., 1994) and exhibited antigen-processing and antigen-presenting capabilities similar to monocytes (Chesney et al., 1997). Despite these similarities, fibrocytes were functionally distinct due to their pronounced role in extracellular matrix deposition and their involvement in harmonizing both inflammatory and reparative phases of wound healing (Chesney et al., 1998). Further research has elucidated key signaling pathways governing fibrocyte differentiation and migration, shedding light on their recruitment to wound sites and their dynamic role in tissue repair (Abe et al., 2001). Notably, emerging evidence suggests that fibrocytes exhibit mesenchymal stem-cell-like properties, raising the possibility that they contribute not only to matrix deposition but also to broader regenerative processes, including multipotent differentiation and tissue remodeling (Bellini and Mattoli, 2007).

While the role of fibrocytes in tissue repair and fibrosis remains an active area of investigation, their potential as a primary cell source for reprogramming into induced pluripotent stem cells (iPSCs) remains largely unexplored. Furthermore, in non-rodent and nonhuman species such as cattle, fibrocyte culture has not been previously attempted. Unlike traditional sources such as skin fibroblasts or embryonic cells, fibrocytes offer a distinct advantage as a readily accessible, diploid somatic cell population that can be isolated from peripheral blood. This enables minimally invasive collection, making fibrocytes an ideal cell source for field applications where aseptic sampling is critical. Compared to skin biopsies, which require specialized handling and pose a higher risk of microbial contamination, blood can be collected via simple venipuncture and either immediately processed or transported for culture under controlled conditions, allowing for the establishment of a stable cell population suitable for reprogramming.

Building on our previous work in generating and sustaining bovine iPSCs from fibroblasts (Pillai et al., 2019; Green et al., 2015), we examined whether bovine fibrocytes could provide an alternative source for iPSC reprogramming. In this study, we define a reproducible workflow for fibrocyte derivation and propagation from Holstein cattle, characterize their transcriptional features, identify signaling programs associated with improved expansion, and test their proof-of-principle capacity to generate iPSCs. Together, these studies position bovine fibrocytes as an accessible, expandable, and reprogrammable primary cell source for livestock stem cell biology, genetic preservation and reproductive biotechnology.

## RESULTS

### Derivation of fibrocytes from bovine peripheral blood

To establish a reproducible method for deriving fibrocytes from adult cattle, nucleated cells obtained from 10 ml of peripheral blood were cultured under adherent conditions in fibroblast medium (Fb) (Fig. 1). After non-adherent cells were rinsed off at 48 h, the remaining population was maintained under daily Fb replacement. During the first 4 days, cultures were dominated by small, phase-bright round cells showing minimal adherence. By Day 7, an increasing number of adherent cells formed discrete microcolonies, and elongated spindle-shaped cells began to appear among residual round cells (Fig. 1B). By Day 10, two prominent morphotypes were consistently observed: rounded, refractile cells with smooth contours and elongated bipolar cells with tapered ends and fine cytoplasmic extensions. These morphological transitions were indicative of a progressive adaptation of adherent leukocytes toward a fibroblast-like phenotype consistent with fibrocyte differentiation. Time-lapse imaging captured this process in real time (Movie 1), showing fibrocyte emergence from a colony-forming nucleation site, followed by outward proliferation and migration across the gelatin-coated surface.

**Fig. 1.**
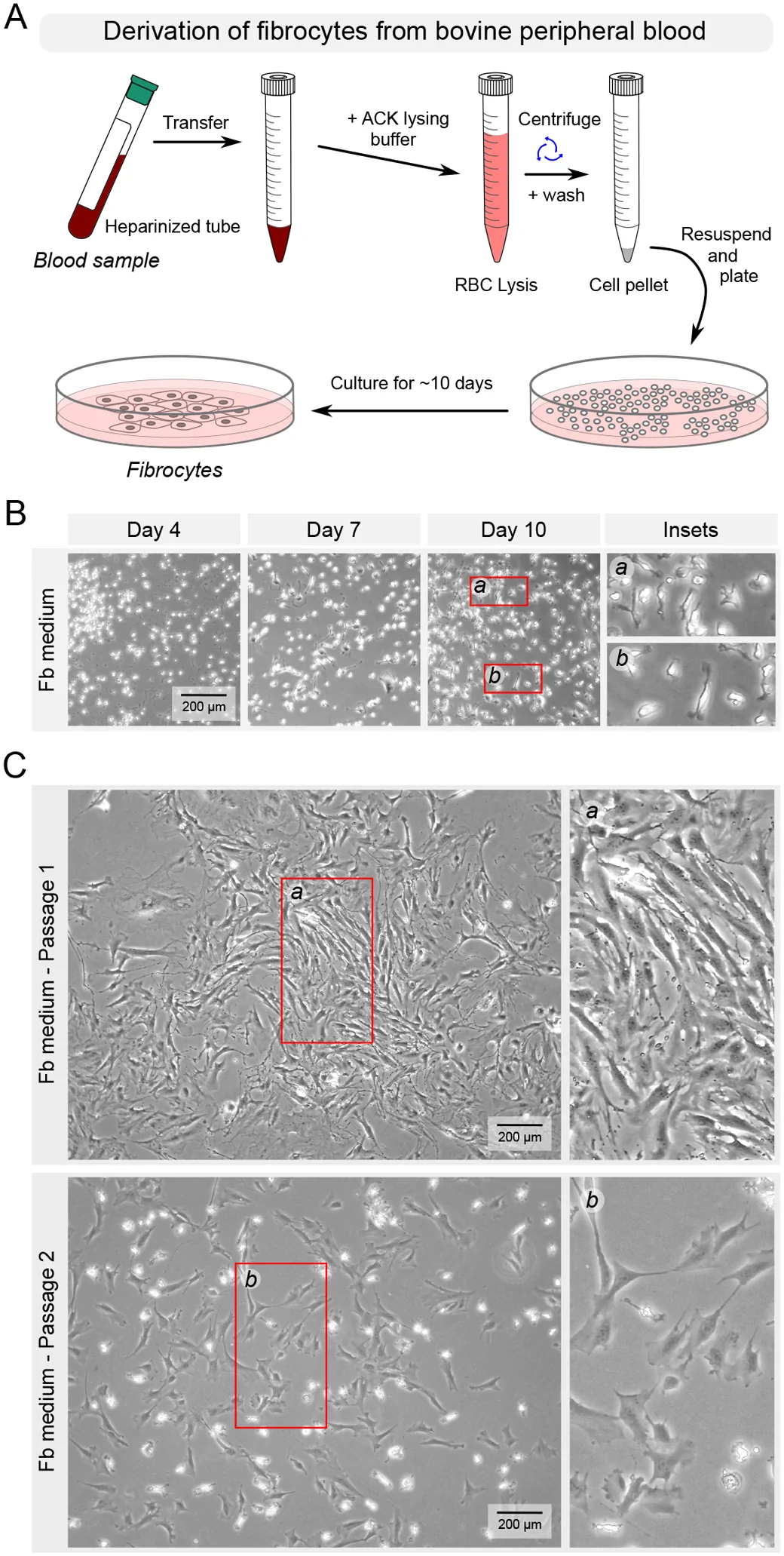
**Derivation of fibrocytes from bovine peripheral blood and morphological characterization.** (A) Workflow outlining the derivation of fibrocytes from bovine peripheral blood. Whole blood collected from the caudal vein was subjected to red blood cell lysis and washing steps. The resulting nucleated cell fraction was plated on gelatin-coated dishes in Fb. Non-adherent cells were removed after 48 h, and adherent cells were expanded with daily medium replacement. Cultures were maintained until fibrocyte growth became evident and continued for approximately 10 days before the first passage. (B) Representative phase-contrast images showing progressive cellular changes during early culture in Fb. At Day 4, the culture consisted predominantly of small, phase-bright round cells with limited adherence. By Day 7, adherent microcolonies emerged, and elongated spindle-shaped cells began to appear among residual round cells. By Day 10, two distinct morphologies were observed: rounded, refractile cells with smooth borders and elongated bipolar cells with tapered ends and thin cytoplasmic extensions. (C) Morphological progression during passaging in Fb. Passage 1 cultures exhibited dense growth of elongated spindle-shaped cells aligned in parallel arrays and whorls. Passage 2 cultures at a lower density displayed greater heterogeneity, with stellate and triangular cells possessing broad lamellipodia and fine filopodial extensions interspersed among slender bipolar cells. Images in B and C are representative of fibrocyte derivations from three independent animals.

Upon passaging, cultures demonstrated further morphological stabilization. Passage 1 cells proliferated rapidly, forming dense formations of elongated spindle-shaped cells organized in parallel streams and whorled arrangements (Fig. 1C). At lower seeding density (Passage 2), cultures became more heterogeneous, displaying stellate and triangular cells with broad lamellipodia and filopodial processes interspersed among slender bipolar cells. Together, these observations documented the reproducible emergence and propagation of fibrocytes from bovine peripheral blood under standard Fb culture conditions.

### Distinct functional identity of bovine fibrocytes

To define the molecular characteristics that distinguish fibrocytes from fibroblasts, we performed RNA-sequencing (RNA-seq) analysis followed by principal component analysis (PCA) and differential expression profiling. PCA revealed clear segregation of the two cell types along the first principal component, which captured the majority of gene expression variance (Fig. 2A). Samples from three independent animals clustered tightly within each group, indicating high within-group consistency and robust separation between fibrocyte and fibroblast transcriptomes.

**Fig. 2.**
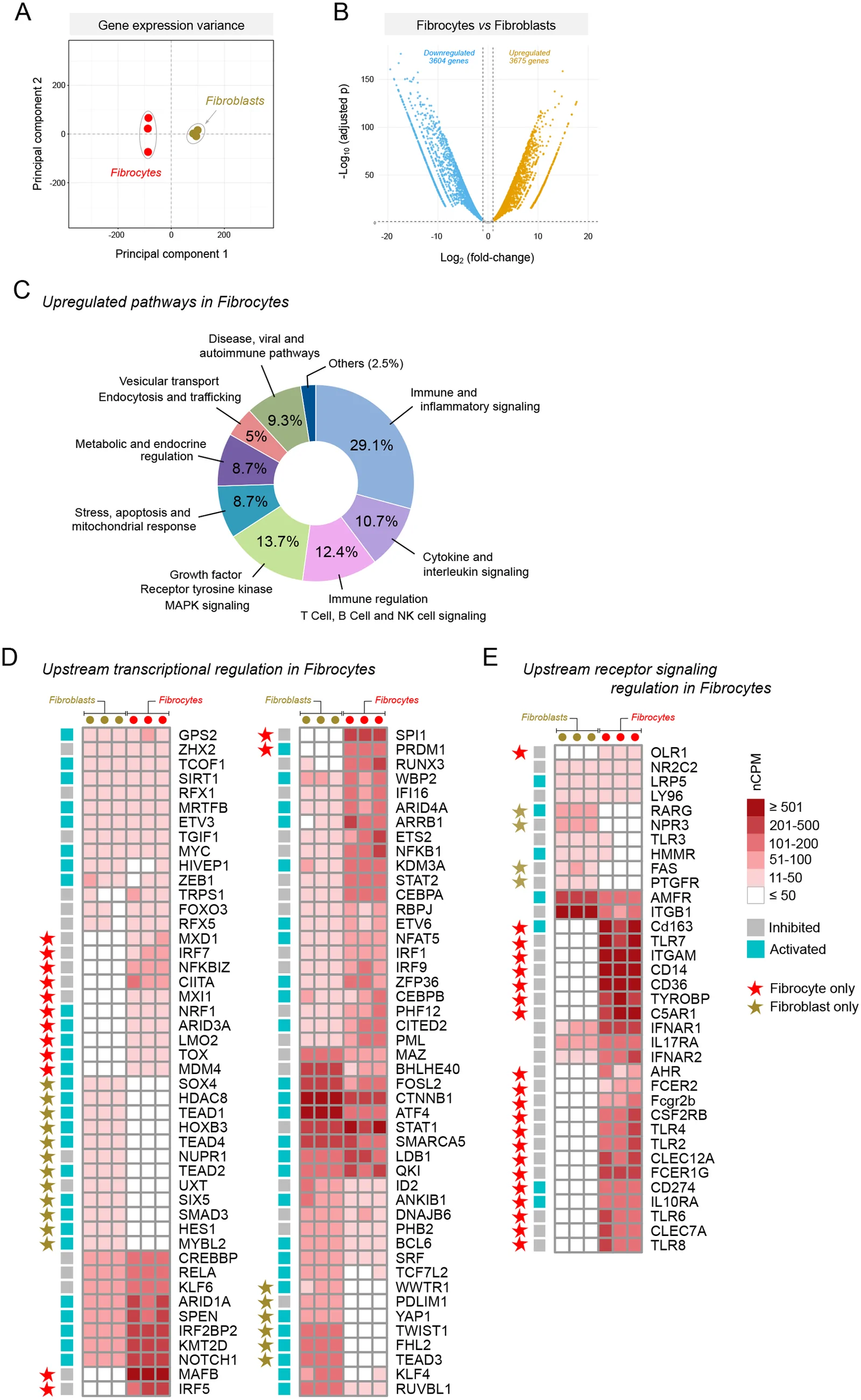
**Transcriptomic distinction and functional enrichment of fibrocytes compared to fibroblasts.** (A) PCA of RNA-seq data showing separation of fibrocytes and fibroblasts based on global gene expression variance. Each point represents an independent animal sample. (B) Volcano plot comparing differential gene expression between fibrocytes and fibroblasts. A total of 3675 genes were significantly upregulated, and 3604 genes were downregulated in fibrocytes (adjusted *P*<0.05). (C) Functional enrichment analysis of genes upregulated in fibrocytes identifying major biological processes and pathways. Prominent categories included immune and inflammatory signaling, cytokine and interleukin signaling, growth-factor-mediated MAPK signaling, metabolic and endocrine regulation, and stress and mitochondrial response pathways. (D) Upstream transcriptional regulators differentially expressed between fibrocytes and fibroblasts. Heatmaps represent nCPM for key transcription factors predicted to drive fibrocyte-specific gene expression. (E) Upstream receptor signaling regulators in fibrocytes. Heatmap displays nCPM values for receptors and signaling molecules differentially expressed between fibrocytes and fibroblasts, highlighting pathways related to immune recognition, cytokine and growth factor signaling, and cellular communication. For both D and E, regulators uniquely expressed in fibrocytes or fibroblasts are indicated by different stars. Color-coded boxes indicate the predicted activation or inhibition status of the upstream regulation.

Differential expression analysis identified extensive remodeling of the fibrocyte transcriptome, with 3675 genes significantly upregulated and 3604 genes downregulated relative to fibroblasts [adjusted *P*<0.05; |log_2_ fold change (log_2_FC)|≥1; Fig. 2B]. Functional enrichment of the upregulated genes revealed dominant representation of immune and inflammatory signaling networks (29.1%), including cytokine, interleukin, and interferon pathways (10.7%), as well as T-, B-, and NK-cell signaling modules (12.4%; Fig. 2C). Additional enriched categories included growth-factor-mediated mitogen-activated protein kinase (MAPK) signaling (13.7%), metabolic and endocrine regulation (8.7%), vesicular trafficking and endocytosis (5%), stress- and mitochondria-related response pathways (8.7%), and disease, viral and autoimmune pathways (9.3%). These findings indicate that fibrocytes maintain a hybrid transcriptional program integrating immune-effector and mesenchymal regulatory functions.

Upstream regulator analysis revealed coordinated activation of transcriptional and receptor-mediated signaling networks that define fibrocyte identity (Fig. 2D,E). Among transcriptional regulators, a fibrocyte-restricted set, comprising SPI1 (PU.1), IRF5, IRF7, NFKBIZ, PRDM1 (BLIMP1), CIITA, MXD1, MXI1, NRF1, MAFB, ARID3A, LMO2, TOX, and MDM4, was predicted to drive key fibrocyte-enriched gene modules. These regulators collectively represent inducible inflammatory and antiviral control (IRF5/IRF7/NFKBIZ/SPI1), antigen-presenting potential (CIITA), hematopoietic lineage memory (ARID3A/LMO2/TOX), and metabolic-stress adaptation (MXD1/MXI1/NRF1/MAFB).

Complementing this transcriptional framework, receptor-level analyses identified a coordinated upregulation of immune and cytokine receptors, including TLR2, TLR4, TLR6, TLR7, TLR8, FCER1G/2, IL10RA, CD14, CD163, C5AR1, and CD274, as well as adhesion and signaling molecules such as ITGAM, CD36, TYROBP, and FAS (Fig. 2E). Together, these receptor and transcriptional networks define a fibrocyte-specific regulatory system that integrates immune sensing, stress adaptation, and reparative signaling, distinguishing fibrocytes as a molecularly distinct somatic lineage rather than a fibroblast variant. All differentially expressed genes (DEGs) and curated gene lists corresponding to each enrichment, upstream regulator, and receptor analysis shown in Fig. 2 are provided in Dataset 1.

### Fibrocyte medium (FbC) promotes stable propagation of blood-derived fibrocytes

Because fibrocytes share features of both mesenchymal and hematopoietic lineages, we developed a modified culture formulation, the FbC, designed to sustain a basal state while maintaining cellular plasticity. FbC incorporates dexamethasone, ascorbic acid, platelet-derived growth factor BB (PDGF-BB), epidermal growth factor (EGF), and small-molecule inhibitors (A83-01 and CHIR99021) to both support mesenchymal expansion and inhibit immune activation that might trigger spontaneous senescence. When nucleated peripheral blood cells were cultured in FbC, small, phase-bright round cells initially dominated the culture. By Day 7, adherent microcolonies emerged, and by Day 10, elongated spindle-shaped fibrocytes became prevalent, displaying organized parallel alignment (Fig. 3A). Upon passaging, fibrocytes retained a uniform spindle morphology and proliferated rapidly with consistent growth across passages, indicating that FbC supported a stable and self-sustaining fibrocyte population (Fig. 3B).

**Fig. 3.**
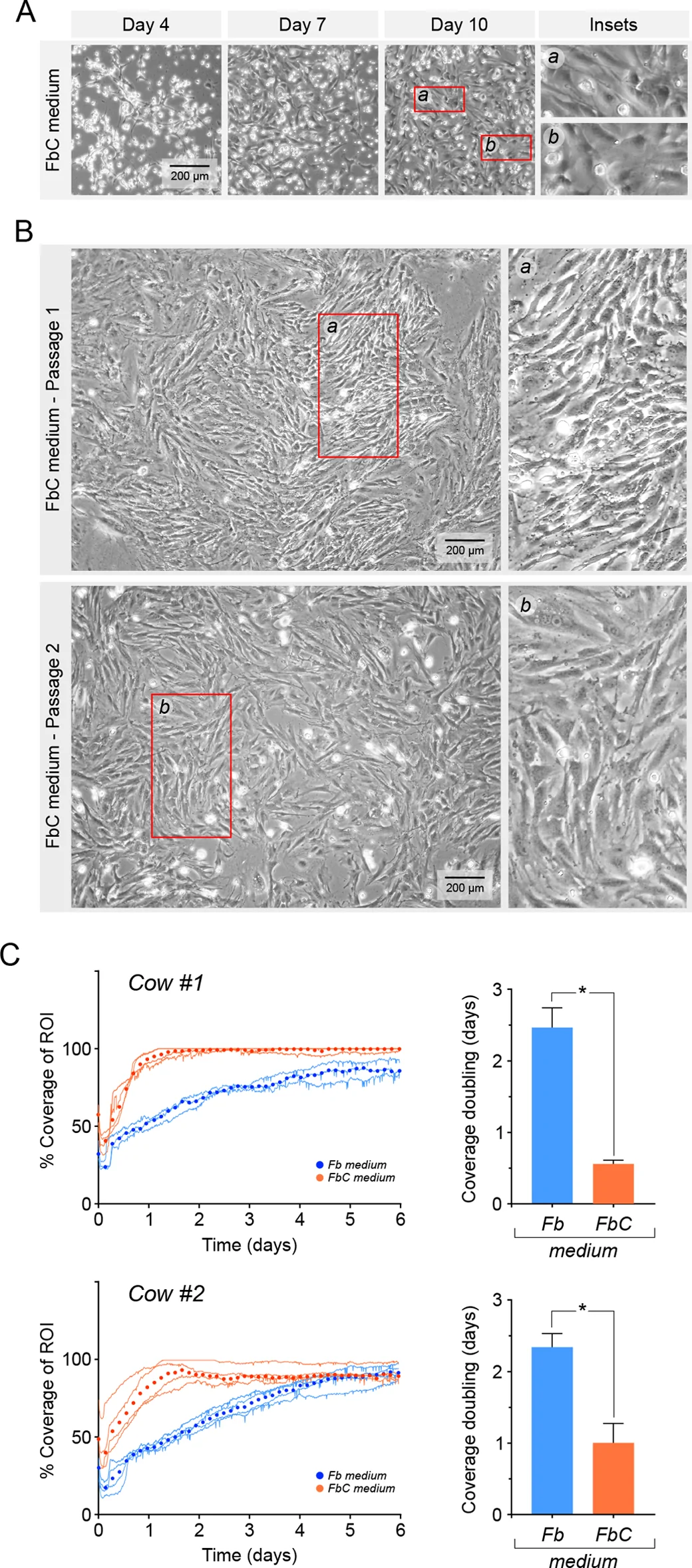
**Derivation and growth kinetics of bovine fibrocytes cultured in FbC.** (A) Sequential phase-contrast images showing the emergence of fibrocytes from bovine peripheral blood in FbC. Small, phase-bright round cells initially dominated the culture, giving rise to adherent microcolonies by Day 7 and elongated spindle-shaped fibrocytes by Day 10, indicative of progressive differentiation and adaptation to adherent growth. Images are representative of fibrocyte derivations from three independent animals. (B) Representative morphology of passaged fibrocytes maintained in FbC showing uniform spindle shape, organized parallel alignment, and sustained proliferation across passages, consistent with stable expansion under these conditions. (C) Growth kinetics comparing fibrocytes cultured in Fb and FbC. Fibrocytes maintained in FbC exhibited significantly faster coverage of the region of interest and a significantly shorter coverage doubling time, indicating that FbC supports more robust fibrocyte expansion. Traces in the growth curve represent independent replicates, and data points indicate median values (*n*=2 animals, Cow #1 and Cow #2). Bar graphs show mean±s.e.m. from three independent biological replicates (*P<0.05).

To visualize these differences dynamically, time-lapse imaging was used to capture fibrocyte behavior under both culture conditions. In Fb, fibrocytes exhibited diffuse growth, extensive migration, and morphological heterogeneity characterized by broad lamellipodia and frequent shape transitions (Movie 2). In contrast, fibrocytes cultured in FbC displayed reduced motility, earlier attachment, and coordinated elongation, followed by rapid mitotic activity and organized palisading proliferation (Movie 3). These live-cell recordings underscore the stabilizing effects of FbC, revealing a shift from migratory adaptation in Fb to sustained, patterned growth in FbC.

Quantitative cell coverage kinetics further substantiated these observations in that fibrocytes cultured in FbC significantly expanded compared to those maintained in Fb (Fig. 3C). Due to differing cell migration and morphologies, cell numbers were difficult to trace. Nevertheless, FbC cultures exhibited characteristics of stable growth and a shorter time to reach 50% coverage (*P*<0.05). These findings demonstrate that the FbC formulation maintains fibrocytes in a mesenchymal-like, plastic phenotype suited for expansion and downstream applications.

### FbC factors cooperatively reprogram fibrocyte transcriptomes

To determine how FbC alters fibrocyte gene expression and functional state, we performed transcriptomic profiling of fibrocytes cultured in Fb versus FbC conditions. PCA revealed separation of samples by medium type, indicating a reproducible transcriptional shift induced by FbC formulation (Fig. 4A). Differential expression analysis identified 2081 genes that were significantly altered between the two conditions (adjusted *P*<0.05, |log_2_FC|>1), with 1140 genes upregulated and 941 genes downregulated in fibrocytes cultured in FbC (Fig. 4B). Hierarchical clustering further confirmed distinct expression signatures, with consistent segregation of FbC-treated fibrocytes across biological replicates (Fig. 4C).

**Fig. 4.**
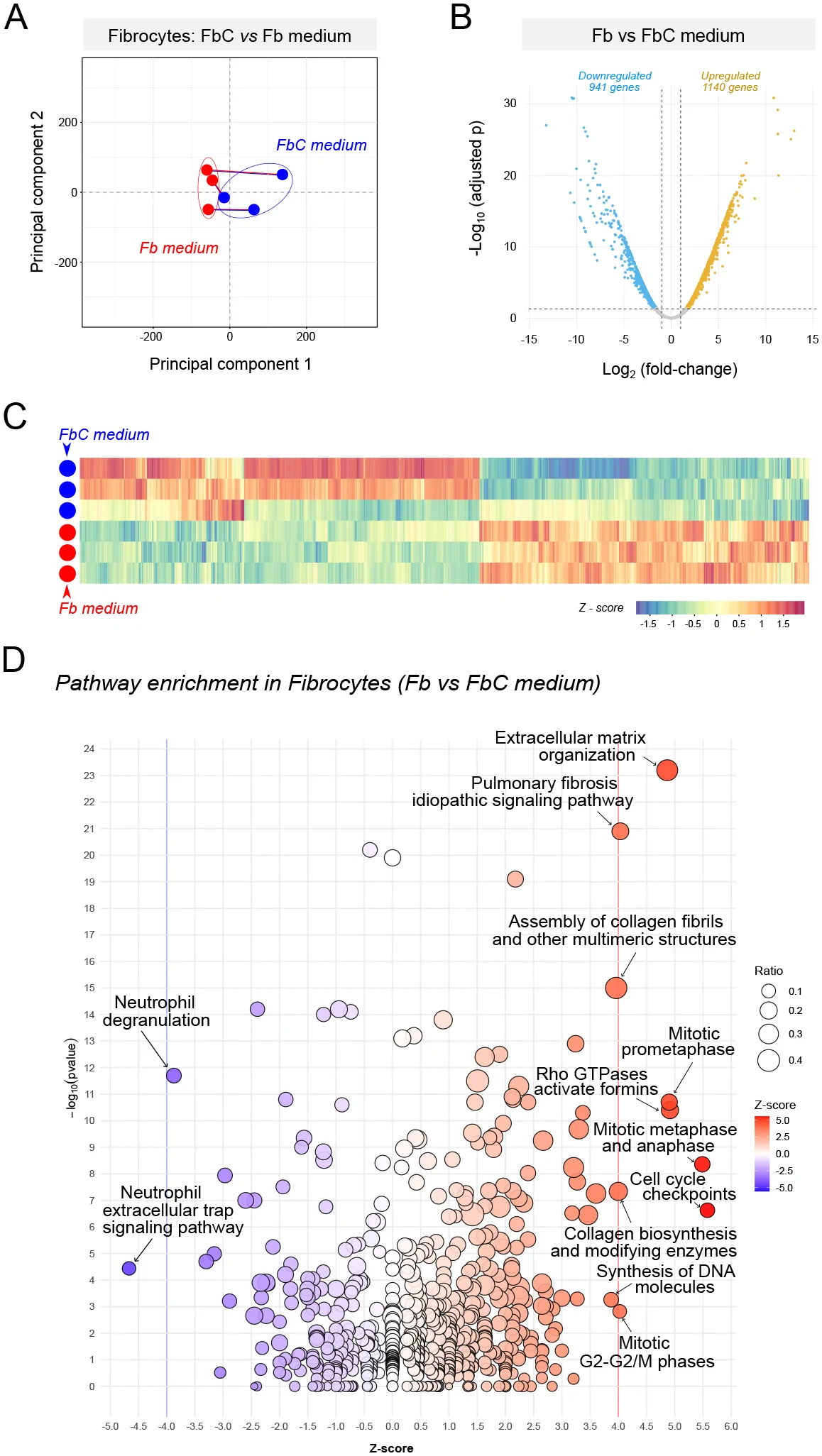
**Transcriptomic effects of FbC on fibrocyte gene expression.** (A) PCA showing distinct clustering of fibrocyte transcriptomes cultured in FbC (blue) versus Fb (red), indicating a strong global transcriptional shift driven by medium composition. (B) Volcano plot of differential gene expression between Fb and FbC conditions, identifying 1140 upregulated and 941 downregulated genes in fibrocytes cultured in FbC (adjusted *P*<0.05, |log_2_FC|>1). (C) Heatmap of DEGs (*z*-score normalized) illustrating consistent transcriptional remodeling of fibrocytes across biological replicates in response to FbC. (D) Pathway enrichment analysis of DEGs showing activation of proliferative, matrix assembly, and cell-cycle pathways (e.g. mitotic progression, collagen biosynthesis, and Rho GTPase signaling) in FbC, with concurrent downregulation of inflammatory response programs. Together, these data demonstrate that FbC promotes a proliferative, matrix-constructive, and reparative transcriptional state in fibrocytes relative to Fb.

Pathway enrichment analysis highlighted a broad reorganization of cellular programs associated with proliferation, cytoskeletal remodeling, and extracellular matrix assembly (Fig. 4D). Upregulated pathways included collagen biosynthesis and modification, assembly of collagen fibrils, mitotic cell-cycle progression, DNA replication, and Rho GTPase-regulated cytoskeletal dynamics, indicative of enhanced matrix production and proliferative activity. Conversely, pathways associated with inflammatory signaling were significantly downregulated, suggesting suppression of immune effector functions.

Upstream regulator-driven gene signatures revealed that each FbC component, dexamethasone, TGFB1 inhibition, EGF, and GSK3β inhibition, exerted a distinct yet highly interconnected transcriptional influence on fibrocytes (Fig. 5A). Dexamethasone showed the broadest influence (444 genes), followed by TGFB1 (424 genes), EGF (160 genes), and WNT activation (32 genes). Dexamethasone, TGFB1, and EGF formed the dominant axis of overlap, but WNT displayed dense connectivity to the other three pathways; only 203, 176, 24, and five genes, respectively, were unique to each regulator. Canonical pathway analysis on the genes responsive to the four signaling inputs/perturbations revealed strong activation of proliferative and matrix-constructive pathways (Fig. 5B). Upregulated pathways included mitotic cell-cycle checkpoints, extracellular matrix organization, collagen biosynthesis and fibril assembly, Rho GTPase–formin signaling, and multiple growth-factor-responsive modules such as mesenchymal-epithelial transition factor (MET), PDGF, and GP6 signaling. In contrast, several immune and inflammatory pathways were consistently predicted to be suppressed. These included IL-17F signaling, NOD1/2 signaling, macrophage classical activation, neutrophil degranulation, TREM1 signaling, and neutrophil extracellular trap formation. These changes indicated that the FbC attenuates inflammatory activation while favoring reparative and proliferative fibrocyte states. All DEG tables and curated gene sets corresponding to the medium comparison and the individual FbC component analyses shown in Figs 4 and 5 are provided in Dataset 2.

**Fig. 5.**
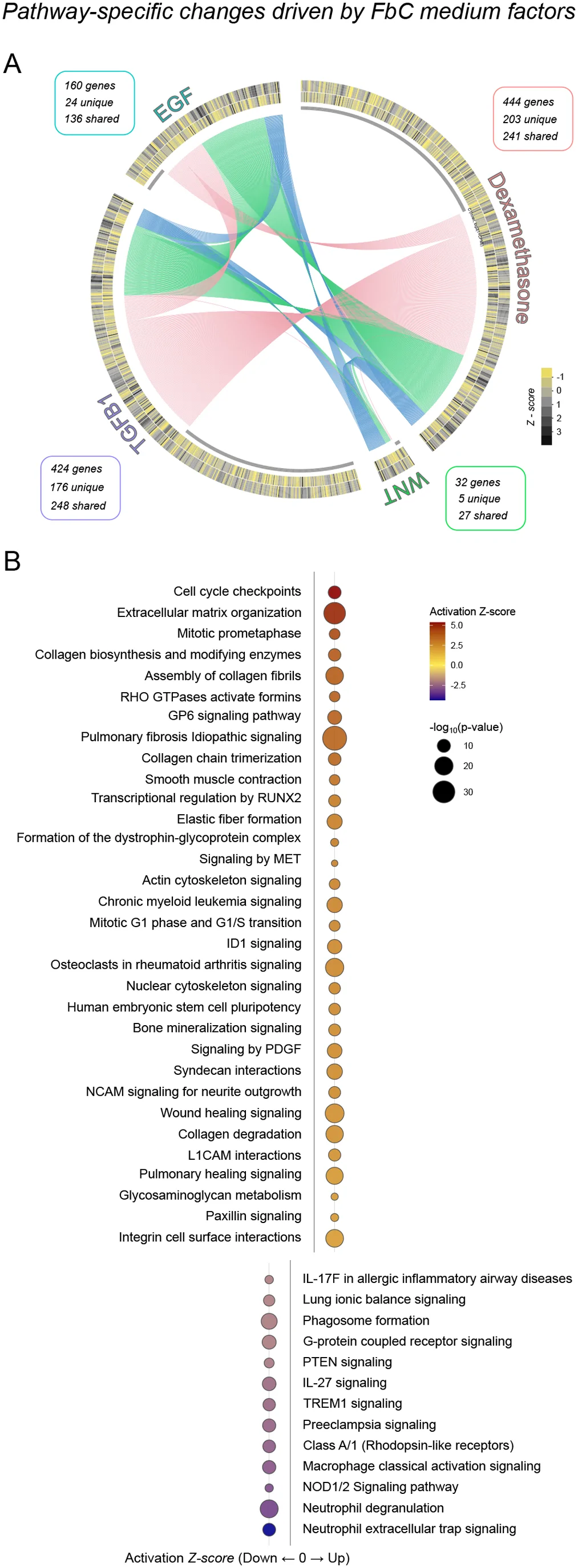
**Pathway-specific transcriptomic modulation by FbC factors.** (A) Circos diagram depicting the extent and overlap of gene expression changes induced by dexamethasone, TGFB1, EGF, and WNT activation in bovine fibrocytes cultured in FbC versus Fb. Outer tracks show scaled expression values (z-scores) for all DEGs associated with each factor. Chord connections indicate shared gene responses, with color coding proportional to the degree of overlap: pink for genes shared by two additives, green for genes shared by three, and blue for genes common to all four additives. Dexamethasone regulated the largest gene set, followed by TGFB1, EGF and WNT. (B) Canonical pathway enrichment analysis showing pathways significantly affected by dexamethasone, TGFB1, EGF, and WNT signaling in fibrocytes. Bubble size corresponds to −log_10_ (*P*-value), and bubble color denotes activation state. The combined actions of these factors strongly activated proliferative and matrix-constructive programs, while suppressing inflammatory and innate-immune pathways.

### Fibrocyte-specific functional gene networks reveal integrated immune and reparative programs

To define the molecular architecture underlying fibrocyte identity, we mapped genes uniquely expressed in fibrocytes cultured in both Fb and FbC but largely absent or undetectable in fibroblasts. High-confidence protein–protein interaction networks were constructed and curated into functional themes reflecting coherent biological processes (Fig. 6). Distinct, interconnected modules emerged, revealing that fibrocytes integrate immune signaling, antigen-processing, and reparative programs within a unified transcriptional framework. Prominent networks included chemokine and cytokine receptors, Nuclear Factor kappa-light-chain-enhancer of activated B cells (NF-κB)/MAPK modulators, and G-protein coupled receptor (GPCR)/lipid mediator signaling, reflecting the capacity for dynamic inflammatory and paracrine communication. Additional clusters representing antigen presentation and processing, complement system, and costimulatory or immune-synapse components highlighted fibrocytes' immunoregulatory potential and antigen-presenting capacity. Modules associated with monocyte/scavenger identity and efferocytosis, together with adhesion/integrin signaling and pattern-recognition receptors, underscored fibrocytes' phagocytic and matrix-interactive functions. A prominent transcriptional regulation cluster encompassing multiple immune regulators linked to Type I interferon and antiviral response networks suggested a strong innate defense axis characteristic of leukocyte/monocyte-derived populations. Finally, smaller but consistent modules related to DNA repair and genome stability implied sustained proliferative potential and resilience under culture adaptation.

**Fig. 6.**
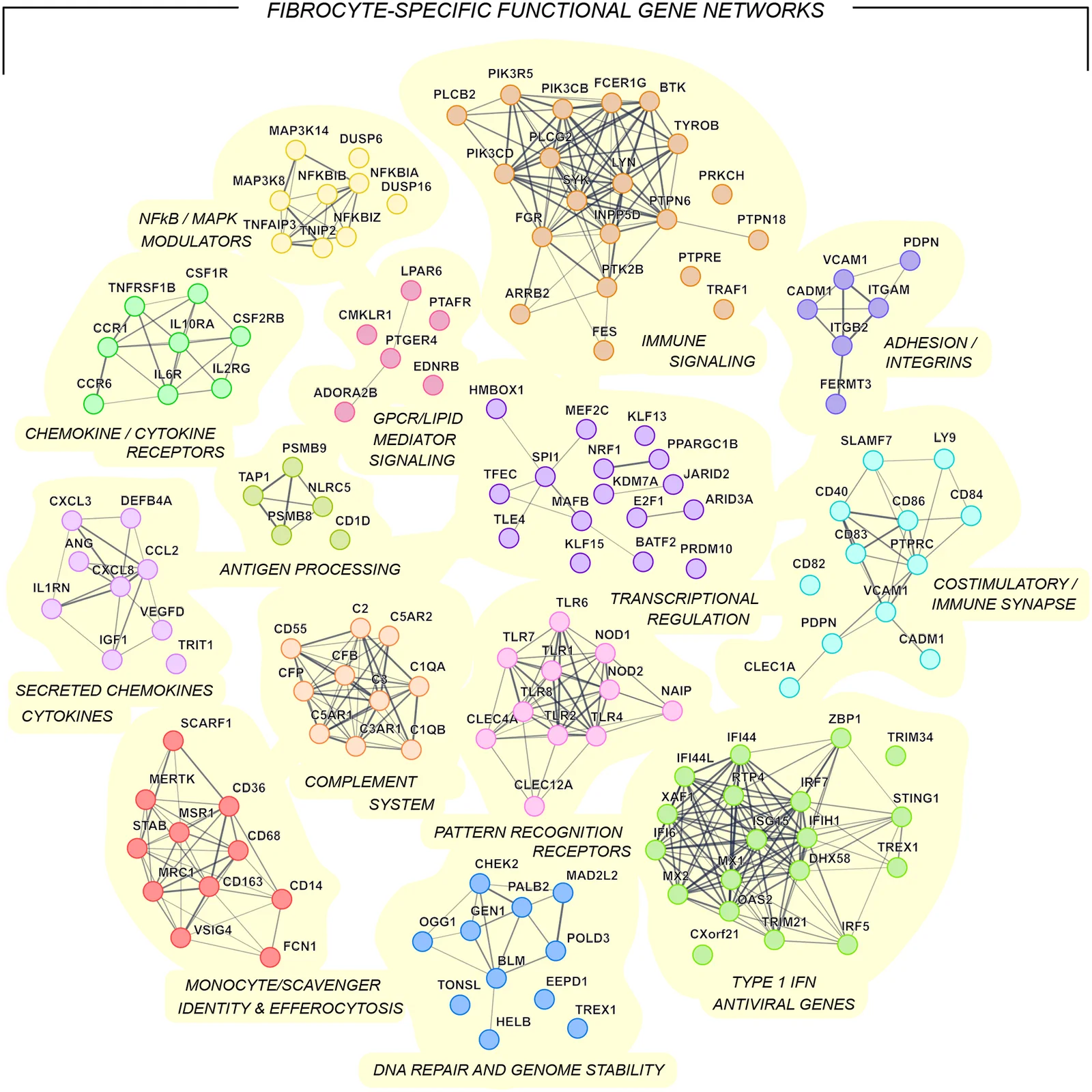
**Fibrocyte-specific functional gene networks.** Functional major gene networks selected from genes uniquely expressed in fibrocytes cultured in both Fb and FbC and largely absent in fibroblasts. High-confidence protein–protein interaction maps were generated and organized into curated functional themes to reveal the dominant molecular programs defining fibrocyte identity. Major network themes include chemokine and cytokine receptors, secreted cytokines, NF-κB/MAPK modulators, GPCR/lipid mediator signaling, antigen presentation and processing, complement system, monocyte/scavenger identity and efferocytosis, pattern-recognition receptors, Type I interferon and antiviral genes, transcriptional regulation, immune signaling, adhesion/integrins, costimulatory and immune-synapse components, and DNA repair/genome stability pathways. Together, these networks outline the coordinated immunoregulatory and reparative signature that distinguishes fibrocytes from fibroblasts.

### Induction of pluripotency in bovine fibrocytes

To determine whether bovine fibrocytes can be reprogrammed to a pluripotent state, fibrocyte cultures established in FbC were subjected to lentiviral transduction using a polycistronic OSKM+LT (Fig. 7A). Following infection, fibrocytes were maintained under feeder-free conditions for 1 week before transfer onto irradiated mouse embryonic fibroblast (MEF) feeders and transition to GMTi medium (Pillai et al., 2021) to facilitate complete reprogramming. Distinct morphological transitions were observed during the reprogramming process (Fig. 7B). By Day 7, fibrocytes formed somewhat dense, proliferative clusters of small, rounded cells that progressively condensed into tightly packed colonies by Day 15. These colonies displayed smooth, dome-shaped borders and high refractility, consistent with a pluripotent morphology. Upon passaging, established fibrocyte-derived colonies exhibited stable growth as compact, multilayered structures with defined colony edges and a high nucleus-to-cytoplasm ratio (Fig. 7C). Alkaline phosphatase (ALP) staining confirmed robust activity in multiple colonies derived from independent cultures, consistent with acquisition of a pluripotency-associated phenotype (Fig. 7D).

**Fig. 7.**
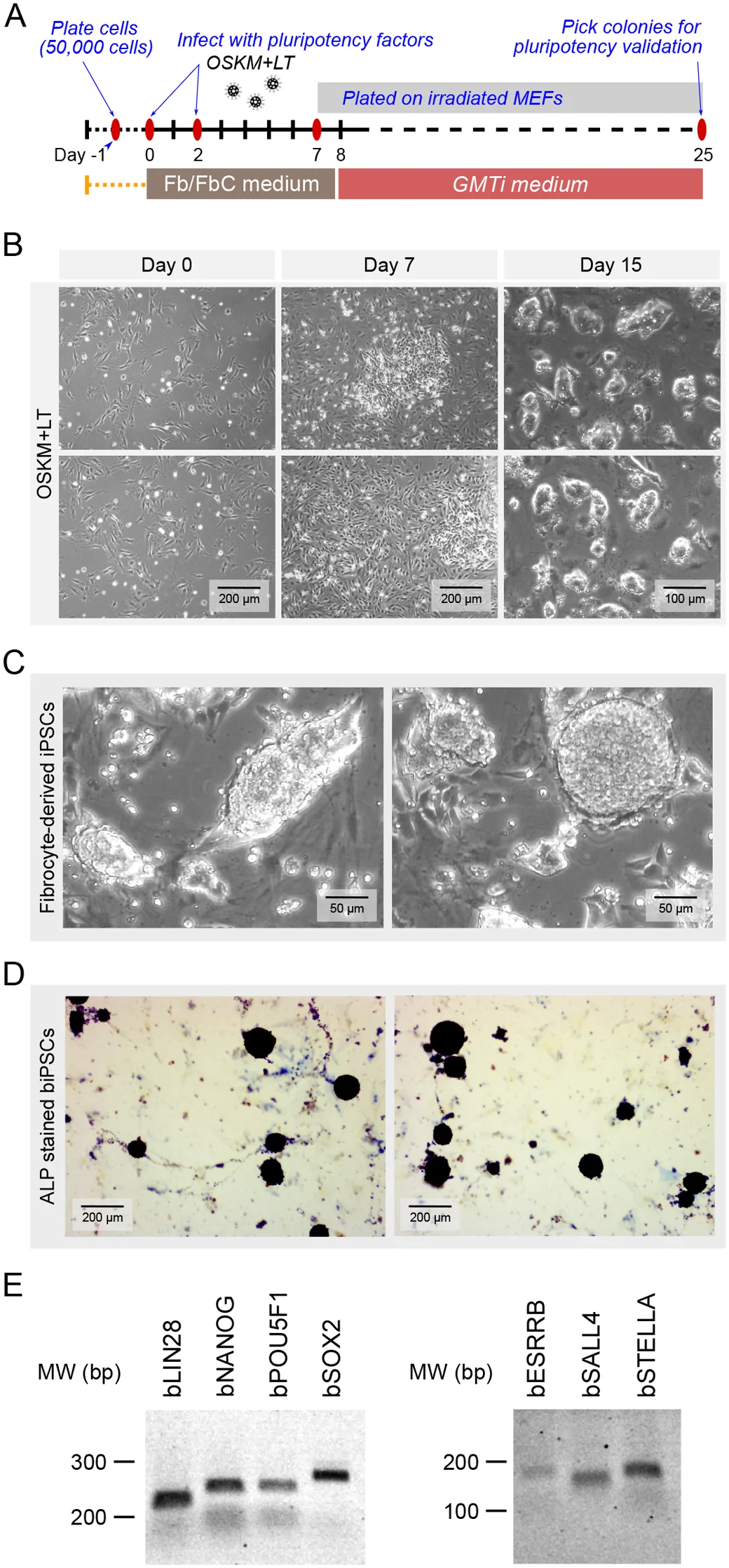
**Induction of pluripotency in bovine fibrocytes.** (A) Experimental timeline for reprogramming fibrocytes to iPSCs. Fibrocytes were plated (Day −1) in FbC, transduced twice with OSKM+LT (Days 0 and 2), transferred to irradiated MEF feeders (Days 7 and 8), and switched to GMTi medium to facilitate reprogramming and sustenance. Putative colonies were picked for propagation and validation within Days 18-25. (B) Representative phase-contrast images from two independent reprogramming experiments showing progressive morphological changes during reprogramming of fibrocytes at Days 0, 7, and 15 after infection. Compact, dome-shaped colonies resembling iPSCs emerged by Day 15. Two representative replicate cultures are shown. (C) High-magnification views showing the morphology of established fibrocyte-derived iPSC colonies after passaging, with characteristic compact, refractile colony borders typical of bovine iPSCs. (D) ALP staining of bovine iPSC colonies generated from fibrocytes, showing robust ALP activity in multiple colonies across two independent plates. Colonies displayed intense staining characteristic of pluripotent stem cells. (E) Endogenous pluripotency gene expression in fibrocyte-derived bovine iPSCs by RT-PCR. The resulting amplicons are presented for core and naïve-associated markers (e.g. bovine POU5F1/OCT4, SOX2, KLF4, ESRRB, SALL4, and STELLA/DPPA3), with size ladders indicated in bp.

To validate endogenous activation of the pluripotency network, reverse transcriptase PCR (RT-PCR) analysis was performed on fibrocyte-derived bovine iPSCs (Fig. 7E). Expression of core pluripotency markers POU5F1/OCT4, SOX2, and KLF4, as well as naïve-associated factors ESRRB, SALL4, and STELLA/DPPA3, were detected in all tested colonies. These results demonstrate that bovine fibrocytes, when cultured in optimized FbC and reprogrammed under GMTi conditions, can give rise to iPSCs exhibiting both morphological and molecular hallmarks of pluripotency.

## DISCUSSION

Circulating somatic cells that acquire reparative or mesenchymal-like phenotypes have broadened our understanding of cellular plasticity in adult tissues (Herzog and Bucala, 2010; Keeley et al., 2009). Fibrocytes are a leukocyte-derived population associated with injury responses, extracellular matrix deposition, inflammation resolution, and tissue remodeling (Reinhardt and Breuer, 2021). Yet their molecular identity and lineage relationships remain incompletely defined, particularly in non-rodent and nonhuman systems. This gap is especially evident in livestock, where fibrocytes have not been derived or evaluated as a field-accessible somatic cell source. Defining bovine fibrocytes at the molecular level is therefore important both for understanding their biology and for assessing their utility as a blood-derived cell type for pluripotency induction.

Fibrocytes occupy a distinctive position between hematopoietic and mesenchymal states, consistent with their reported roles in immune regulation and tissue repair (Reilkoff et al., 2011). Human and murine fibrocytes share a composite hematopoietic–mesenchymal phenotype, including expression of CD45/CD11b together with collagen and chemokine-receptor programs, although individual markers such as CD34, CD45, and chemokine receptors vary with species, culture conditions, and differentiation state (Reinhardt and Breuer, 2021; Reilkoff et al., 2011; Pilling et al., 2009a,b). Bovine blood-derived fibrocytes showed a similar mixed molecular profile, integrating immune, reparative, and metabolic programs. Chemokine and GPCR modules enriched in bovine fibrocytes aligned with prior evidence that leukocyte-derived precursors traffic to sites of damage through chemokine-mediated recruitment involving CCR1- and CXCR4-associated signaling pathways (Abe et al., 2001; Liu et al., 2017; Scholten et al., 2011; Eis et al., 2004). Matrix-associated programs were also prominent, including upregulation of COL1A1, COL3A1, FN1, MMP9, and SERPINE1 and enrichment of collagen biosynthesis and fibril assembly pathways, consistent with Type I collagen and fibronectin production described in fibrocytes within injured tissues (Chesney et al., 1998; Blakaj and Bucala, 2012). In parallel, the expression of interleukin 6 (IL6), tumor necrosis factor (TNF), and C-C motif chemokine ligand 2 (CCL2) reflected cytokine and chemokine programs implicated in local immune modulation and leukocyte recruitment (Ekert et al., 2011; Galligan and Fish, 2013). Bovine fibrocytes also expressed major histocompatibility complex (MHC)-II-associated and costimulatory genes (CD80, CD86), consistent with antigen-presentation programs previously demonstrated in human fibrocytes (Pilling et al., 2009a). Enrichment of MAPK and phosphoinositide 3-kinase (PI3K) signaling, together with expression of receptors such as CSF1R (Rojo et al., 2019), further suggests responsiveness to paracrine cues such as transforming growth factor-β (TGF-β), PDGF, and vascular endothelial growth factor (VEGF) associated with proliferation, survival, and tissue remodeling (Hartlapp et al., 2001; Ferrari et al., 2009). Moreover, prior evidence that fibrocytes can differentiate into osteogenic, chondrogenic, adipogenic and myogenic lineages (Choi et al., 2010; Hong et al., 2007) aligns with the metabolic and developmental plasticity suggested by the bovine fibrocyte transcriptome. Collectively, these findings support a model in which bovine fibrocyte derivation *in vitro* captures a hematopoietic cell state that integrates immune, matrix-associated, and plasticity-related programs, providing a biological rationale for leveraging these cells as a plastic somatic platform for reprogramming.

Upstream regulator analysis identified a fibrocyte-restricted transcriptional network associated with SPI1, IRF5/IRF7, NFKBIZ, PRDM1, CIITA, and MAFB, defining a core regulatory axis characteristic of myeloid-derived cells. These transcription factors collectively delineate a hematopoietic control layer that persists in fibrocytes despite acquisition of mesenchymal and reparative features. SPI1 (PU.1) serves as a master regulator of myeloid specification, maintaining expression of receptors such as CSF1R, CD14, and integrins that underpin mononuclear phagocyte identity and immune competence (McKercher et al., 1996; Scott et al., 1994). The interferon regulatory factors IRF5 and IRF7 orchestrate inducible inflammatory and antiviral programs downstream of toll-like receptor signaling (Honda and Taniguchi, 2006; Krausgruber et al., 2011), while NFKBIZ (IκBζ) acts as a noncanonical NF-κB coactivator that controls late-phase cytokine genes including IL6 and TNF (Hörber et al., 2016; Yamamoto et al., 2004). Together, these regulators create an inducible immune-stress axis consistent with the cytokine and chemokine responses observed in fibrocytes. In contrast, PRDM1 (BLIMP1) and MAFB temper proinflammatory signaling and guide monocyte-to-macrophage maturation, reinforcing a transcriptional switch toward resolution and repair. PDRM1, though broadly expressed across hematopoietic lineages, functions in monocytes and macrophages as a transcriptional repressor that limits inflammatory cytokine production and promotes transition toward resolution (Ulmert et al., 2020). MAFB, a transcription factor downstream of SPI1, promotes monocyte-to-macrophage differentiation and represses proliferative signaling while integrating lipid and anti-inflammatory responses (Kelly et al., 2000). Finally, CIITA, the master transactivator of MHC class II genes, provides a molecular basis for the antigen-presenting potential reflected in fibrocyte expression of MHC II, CD80, and CD86 (Steimle et al., 1993). The coordinated expression of these factors in bovine fibrocytes therefore supports a model in which partial hematopoietic lineage memory is transcriptionally retained.

Optimization of the fibrocyte culture environment – incorporating dexamethasone, ascorbic acid, PDGF-BB, EGF, A83-01, and CHIR99021 – was essential to reduce proinflammatory signaling while promoting growth and preserving lineage plasticity. Conventional Fb supported only limited adherence and expansion, suggesting that the basal conditions failed to maintain the signaling equilibrium permissive to growth. Transcriptomic comparisons between Fb and FbC cultures revealed extensive factor-driven programming across cell cycle, mitotic progression, and cytoskeletal remodeling, accompanied by suppression of inflammatory signaling. Glucocorticoid signaling is known to restrain proinflammatory activity through MAPK inhibition, NF-κB interference, and induction of the phosphatase DUSP1/MKP-1 (Barnes, 1998), providing a mechanistic basis for the dampening of immune programs observed under FbC conditions. Ascorbate supports cellular remodeling and viability by promoting prolyl and lysyl hydroxylase activity and procollagen maturation, while also enhancing TET-dependent DNA demethylation that improves somatic cell reprogramming competence (Chen et al., 2013; Chung et al., 2010; Booth and Uitto, 1981; Hata and Senoo, 1989), consistent with the proliferative, plastic state we detect in FbC cultures. PDGF-BB activates PI3K–AKT and ERK pathways to drive mesenchymal stem cell proliferation, motility, and survival (Zhang et al., 2018; Gharibi et al., 2012), aligning with the cell-cycle and cytoskeletal programs enriched in FbC relative to Fb. In parallel, soluble EGF augments human mesenchymal stem cell (MSC) proliferation while preserving early progenitors and boosting paracrine outputs (Tamama et al., 2010), providing a contextual basis for the growth and pro-angiogenic signaling we observe in FbC cultures. To avoid TGF-β-driven differentiation, the Activin/NODAL/TGFβ pathway inhibitor A83-01 supports expansion of undifferentiated mesenchymal and epithelial progenitors and enhances reprogramming efficiency in somatic cells (Gurung et al., 2015), consistent with the goal of maintaining a reprogrammable fibrocyte state. Finally, CHIR99021 stabilizes β-catenin to activate canonical WNT signaling, a well-established mechanism for sustaining self-renewal and cytoskeletal organization in mesenchymal stem cell systems (Huang et al., 2017; Govarthanan et al., 2020), matching the FbC-induced transcriptional shift toward proliferative, motile phenotypes. Collectively, these component-level activities provide a plausible framework for interpreting the FbC-associated transcriptional shift toward MAPK and PI3K signaling, reduced inflammatory activation, and a growth-permissive fibrocyte state. Because validated bovine fibrocyte protein-marker criteria are not currently established, the present cell identity is defined primarily through convergent morphological, culture and transcriptomic evidence; future protein-level studies will be important for further refining phenotypic criteria for bovine fibrocytes.

The establishment of a proliferative fibrocyte population under FbC conditions provided a biologically advantageous platform for somatic reprogramming. Fibrocytes possess intrinsic features that may lower the epigenetic barrier to pluripotency, including active chromatin states associated with inflammatory signaling, mitochondrial metabolism, and stress adaptation. These characteristics parallel those seen in hematopoietic or myeloid-derived cells that reprogram with relatively high efficiency compared to terminally differentiated fibroblasts (Okabe et al., 2009; Eminli et al., 2009). The retention of hematopoietic transcriptional regulators such as SPI1, IRF5, and PRDM1, together with metabolic and proliferative signatures reinforced by FbC conditions, likely facilitates chromatin accessibility and responsiveness to the OSKM+LT factors used for reprogramming (Pillai et al., 2021). Consistent with this, fibrocytes cultured in FbC transitioned to compact, ALP-positive colonies that expressed endogenous POU5F1, SOX2, KLF4, and naïve pluripotency factors (ESRRB, SALL4, STELLA/DPPA3), supporting acquisition of a pluripotency-associated state. These observations align with prior evidence that optimized mesenchymal or hematopoietic contexts enhance iPSC derivation through PI3K–AKT and WNT signaling, as well as through vitamin-C-driven epigenetic remodeling (Zhang et al., 2014; Wang et al., 2011; Esteban et al., 2010). In this context, bovine fibrocytes represent a naturally accessible, field-adaptable somatic cell type that can be isolated aseptically from peripheral blood and stably propagated without genomic alteration. The proof-of-principle reprogramming of these cells to iPSCs supports the feasibility of generating genetically defined pluripotent lines from valuable or endangered livestock, establishing a foundation for genetic preservation, reproductive biotechnology, and regenerative applications in large-animal systems.

Together, these findings establish a molecular framework defining bovine fibrocyte characteristics and demonstrate their use as a practical somatic cell source for reprogramming. By combining transcriptomic profiling with targeted culture optimization, this study shows that bovine fibrocytes represent an inducible monocyte lineage-associated state with integrated immune, reparative, and metabolic programs that can be expanded *in vitro*. The FbC supports proliferative competence while suppressing inflammatory activation, providing a culture environment that preserves fibrocyte-associated features during expansion. Given their accessibility from peripheral blood, capacity for expansion, and compatibility with reprogramming, bovine fibrocytes provide a field-adaptable somatic cell platform for pluripotency induction in valuable or genetically diverse livestock.

## MATERIALS AND METHODS

### Animals, blood collection and preparation

Whole blood was collected by venipuncture of the caudal vein of Holstein cattle (*Bos taurus*; 1-2 years of age) in heparinized Vacutainer^®^ tubes (BD, Switzerland) at the Cornell University Ruminant Center. Blood was processed immediately within 1-2 h of collection for red blood cell lysis to obtain nucleated cells. For this step, blood was diluted in ammonium-chloride-potassium red blood cell lysis buffer (composition: 150 mM NH_4_Cl, 10 mM KHCO_3_ and 0.1 mM Na_2_EDTA) at a 1:9 ratio and incubated at 37°C for 5 min. The sample was then centrifuged for 5 min at 200×***g***, and the supernatant was removed. This process was repeated once to ensure the resultant pellet was devoid of red blood cells and appeared completely white. The pellet was then resuspended in PBS and centrifuged again as a wash. This final pellet was plated in 0.1% gelatin-coated dishes for cell culture (Fig. 1A). Animal procedures were approved by the Institutional Animal Care and Use Committee of Cornell University. All chemicals were purchased from MilliporeSigma (St. Louis, MO, USA), unless otherwise noted.

### Fibrocyte cell culture

The pellet of nucleated blood cells was resuspended either in a simple medium routinely used for culturing bovine fibroblasts (Pillai et al., 2019), Fb [composition: high-glucose DMEM, 10% fetal bovine serum, penicillin–streptomycin, and nonessential amino acids (Gibco)], or in a modified mesenchymal stem cell medium, FbC [composition: high-glucose DMEM, 10% fetal bovine serum, penicillin–streptomycin, nonessential amino acids, 10 nM dexamethasone, 0.1 mM L-ascorbic acid-2-phosphate, 10 ng/ml PDGF-BB (GoldBio), 10 ng/ml EGF (GoldBio), 0.5 μM A83-01 (Cayman Chemical Company), and 1.5 μM CHIR99021 (Cayman Chemical Company)]. The biological rationale for the FbC components was as follows: dexamethasone to restrain inflammatory activation, ascorbate to support epigenetic remodeling, PDGF-BB and EGF to promote proliferation, A83-01 to limit differentiation, and CHIR99021 to activate canonical WNT/β-catenin signaling. Resuspended cells were plated on 0.1% gelatin-coated cell culture dishes and incubated at 37°C in an atmosphere of 5% CO_2_. After 48 h, the plate was gently washed with PBS to remove non-adherent cells and debris, and fresh medium was provided. Medium was subsequently replaced every 24 h thereafter and observed for colony formation. Once plates were ∼70% confluent, colonies were passaged for experimentation or frozen for future use. Freezing was performed by resuspending cell pellets in 1 ml of freezing medium (90% FBS with 10% DMSO), transferring to cryovials and placing them in freezing containers at −80°C (Thermo Fisher Scientific). After 24 h, vials were moved to liquid nitrogen tanks for long-term storage. Matched samples from the same collection from each cow were cultured in Fb and FbC to compare the different characterizations.

### Proliferation and morphological analysis

Fibrocyte morphology was assessed throughout culture using phase-contrast microscopy. Cells were seeded on 0.1% gelatin-coated culture dishes and maintained under standard conditions. Images were acquired at defined time points using a DM IL LED inverted microscope equipped with an MC190 HD camera (Leica Microsystems). Cell shape, spreading, and adherence characteristics were recorded, and representative fields were imaged at multiple magnifications to document progressive changes in morphology. Bright-field imaging was performed using the Lumaview 720/600 series live-imaging microscope to capture videos of cells cultured from initial peripheral blood draws following red blood cell lysis treatment. The recordings were used to visualize nucleation sites and monitor the evolving cellular morphology and growth dynamics over time, with individual image frames subsequently stitched together to produce a continuous time-lapse video. Percentage of cell coverage was quantified from calibrated images acquired using a zenCELL Owl live-cell imager (innoME GmbH). Images were acquired from randomly selected fields within 24-well plates, in which four wells were allocated to each medium condition (Fb or FbC) for each of two individual cows. Bright-field images were acquired every 10 min in a 24-well plate using a 10× magnification setting on the live-cell imager. The onboard algorithm segments each image by classifying pixels as either ‘cell covered’ or ‘background’ based on contrast thresholds and then computes cell coverage (%) as the ratio of cell-covered pixels to total field area (overlapping cells are not double counted).

For each cow, four wells per medium condition (Fb and FbC) were imaged continuously. The cell coverage (%) values were plotted against time to generate growth curves for each well and medium condition. For visualization and comparison, the median cell coverage across the four replicate wells per condition was plotted as the representative curve. The doubling time (*T_d_*) was determined by selecting the most representative exponential-growth segment of each curve and applying the standard proliferation-rate formula:

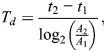
where *A*_1_ and *A*_2_ represent cell coverage at times *t*_1_ and *t*_2_, respectively. Finally, the resulting image sequence from each treatment group was processed and compiled into continuous time-lapse videos to visualize cellular dynamics.

### RNA extraction and RNA-seq

Total RNA was extracted from samples of three individual cows using the RNAqueous Micro Total RNA Isolation Kit (Thermo Fisher Scientific). For each animal, cells were cultured for 10 days at an equivalent seeding density under Fb or FbC conditions. Following this period, the cells were passaged once before RNA extraction. The quantity and purity of the RNA were assessed by spectrophotometry using a NanoPhotometer N60 (Implen), and the integrity of the RNA was confirmed using a Bioanalyzer 2100 (Agilent Technologies). Samples with RNA integrity numbers >8.0 were used for library preparation. RNA-seq libraries were generated using the TruSeq Stranded mRNA Library Preparation Kit (Illumina), incorporating poly-A selection to enrich for mRNA. Libraries were quantified by quantitative PCR and pooled for sequencing on an Illumina NovaSeq X Plus platform to obtain 150 bp paired-end reads. Read quality was evaluated using FastQC (Babraham Bioinformatics). Clean reads were aligned to the *B. taurus* reference genome (ARS-UCD1.2) using STAR, and gene-level counts were obtained with edgeR.

### Transcriptome analysis

Differential expression was performed using pairwise quasi-likelihood comparisons in edgeR, and DEGs were identified using pairwise comparisons with a false discovery rate (FDR)<0.05. For comparative analyses involving primary fibroblasts, publicly available RNA-seq data from the National Center for Biotechnology Information (NCBI) Gene Expression Omnibus (GEO) (GSE61027) (Green et al., 2015) were incorporated and analyzed in parallel using the same normalization and statistical thresholds. This dataset was selected as a well-characterized bovine primary dermal fibroblast transcriptome, providing a representative somatic fibroblast reference. Canonical pathway and upstream regulator analyses were performed using Ingenuity Pathway Analysis (Qiagen). For fibrocyte–fibroblast comparisons, canonical pathways with activation *z*-scores of ≥+2 or ≤−2 were classified as activated or inhibited and grouped into functional biological categories. Pathways were visualized using bubble plots to identify those with strongly activated or suppressed *z*-scores. Upstream regulator analysis identified transcription factors and receptors that are predicted to drive fibrocyte-specific gene expression programs; regulators with |*z*|≥2 and *P*<0.05 were retained. Normalized counts per million (nCPM) for genes encoding these regulators were row scaled and visualized using the pheatmap package with hierarchical clustering to illustrate predicted activation and inhibition patterns. To evaluate transcriptional responses attributable to the components of the FbC, DEGs contributing to the identification of dexamethasone, TGFB1, EGF, and WNT as upstream regulators were extracted and grouped according to regulator assignment. Shared and unique gene sets were determined by intersecting the DEG lists across regulators, yielding discrete categories representing convergent or factor-specific responses. Circos plots were generated using the circlize package to visualize the extent of transcriptional overlap among these regulator-defined gene sets. Fibrocyte-specific functional networks were generated from categorized gene lists that were curated and visualized as protein–protein interaction networks using STRING (string-db.org).

### Reprogramming to pluripotency

Reprogramming of bovine fibrocytes was performed using methods previously described (Pillai et al., 2021), with minor modifications. Briefly, fibrocytes were plated (5×10^4^ cells per well) and infected with a human polycistronic lentiviral vector encoding OCT4, SOX2, KLF4, and c-MYC (Sommer et al., 2009; Pillai et al., 2021), together with a lentiviral SV40 large T-antigen vector (Pillai et al., 2021). Lentiviral packaging and production were carried out as described previously (Pillai et al., 2019). The medium was replaced and transferred to irradiated MEF feeders as indicated. The cultures were then maintained in GMTi medium [DMEM/F-12 supplemented with N2, B-27, 1% nonessential amino acids, 1% penicillin–streptomycin, 0.1 mM β-mercaptoethanol, 1.5 μM CHIR99021, 1 μM PD0325901, 0.5 μM A83-01, and 20 ng/ml human leukemia inhibitory factor(hLIF)] for colony expansion and maintenance (Pillai et al., 2021). Colonies exhibiting compact morphology, high nuclear-to-cytoplasmic ratios, and well-defined borders were manually selected and expanded for further characterization.

### ALP detection

Labeling for ALP activity was performed as previously described (Pillai et al., 2019). Briefly, ALP activity was visualized using the Vector Blue AP Substrate Kit (Vector Laboratories) according to the manufacturer's instructions. Reagents were mixed in 10 ml of Tris–HCl buffer (pH 8.5) and added directly to cell culture plates, followed by incubation for 30 min at 37°C in 5% CO_2_. Stained colonies were imaged under transmitted light without contrast using a DM IL LED inverted microscope equipped with an MC190 HD camera (Leica Microsystems).

### RT-PCR

cDNA was synthesized from 2 μg of total RNA using the High-Capacity cDNA Reverse Transcription Kit with MultiScribe™ Reverse Transcriptase (Life Technologies) and Oligo(dT) primers (Biomatik). Reverse transcription was performed in a C1000 Touch thermal cycler (Bio-Rad) under the following conditions: 25°C for 10 min (primer annealing), 37°C for 120 min (cDNA synthesis), and 85°C for 5 min (enzyme inactivation), followed by a hold at 4°C. The resulting cDNA was diluted 1:2 with nuclease-free water and stored at −20°C until further use. Gene-specific primers (Table S1) were subsequently used for PCR amplification of bovine targets. Each 20 μl of PCR contained 1 μl of cDNA template, 0.4 μl of 10 mM deoxynucleoside triphosphates (dNTPs), 1 μl each of 10 μM forward and reverse primers, 0.5 μl of Taq polymerase, 2 μl of 10× PCR buffer, and nuclease-free water to volume.

### Statistical analysis

For quantitative non-sequencing experiments, individual collections or experiments were treated as biological replicates, and replicate wells or fields from the same biological sample were treated as technical replicates. Technical replicates were summarized within each biological replicate before statistical comparison. Comparisons between two conditions were performed using two-tailed paired Student's *t*-tests. Data are presented as mean±s.e.m., and *P*<0.05 was considered statistically significant.

## Supplementary Material



10.1242/biolopen.062486_sup1 Supplementary information

Dataset 1. Transcriptomic distinctions between fibrocytes and fibroblasts. **Tab 1**: Genes differentially expressed between fibrocytes (in Fb medium) and fibroblasts. **Tab 2**: Canonical pathways enriched in fibrocytes compared to fibroblasts. **Tab 3**: Prediction of upstream transcriptional regulation driving the gene expression pattern in fibrocytes. **Tab 4**: Prediction of upstream receptors driving the gene expression pattern in fibrocytes. **Tab 5**: Fibrocyte-specific gene expression data used to formulate functional gene networks.

Dataset 2. Transcriptomic effects driven by fibrocyte medium (FbC) conditions. **Tab 1**: Genes differentially expressed between fibrocytes in FbC vs Fb medium conditions. **Tab 2**: Canonical pathways enriched in fibrocytes in FbC medium compared to fibrocytes in Fb medium. **Tab 3**: Genes regulated by FbC medium factors: dexamethasone, WNT signaling, TGFB1 inhibition, and EGF signaling.
