# Supplementary material for: Signal-integrated control of immune and reparative programs establishes blood-derived fibrocytes reprogrammable toward induced pluripotency
Source: Biol Open. 2026 Sep 29;15(9):bio062486. doi: 10.1242/bio.062486 (PMC13670359; doi:10.1242/bio.062486)
Supplement: Supplementary file 1 [file biolopen-15-062486-s1.pdf]

Table S1. Primers used for RT-PCR

Gene	Forward Primer	Reverse Primer	Product Size
LIN28A	5'-GGCCGTGGAGTTCACCTTTA-3'	5'-GTGGCAGTTTGCACTCCTTG-3'	194 bp
NANOG	5'-GTGTTTGGTGAACTCTCCTG-3'	5'-GGGAATTGAAATACTTGACAG-3'	308 bp
OCT4	5'-GTTGTGTTTGGAAAGGTGTTC-3'	5'-ACACTCGGACCACGTCTTTC-3'	313 bp
SOX2	5'-AACTCGGAGATCAGCAAGCG-3'	5'-GGTCTGCGAGCTGGTCATAG-3'	444 bp
ESRRB	5'-ATGTTGAAGGAAGGTGTGCG-3'	5'-ATGGCATACAGTTTGTCCGG-3'	179 bp
SALL4	5'-AGAGCGTCAAGTCCAAGTCT-3'	5'-TGGGCTCGGATAAATGTGGA-3'	112 bp
DPPA3	5'-ATCCATCGATGACGCTTCCC-3'	5'-CCGCCTATGGGGTGTTTTCT-3'	172 bp

Dataset 1. Transcriptomic distinctions between fibrocytes and fibroblasts. Tab 1: Genes differentially expressed between fibrocytes (in Fb medium) and fibroblasts. **Tab 2:** Canonical pathways enriched in fibrocytes compared to fibroblasts. **Tab 3:** Prediction of upstream transcriptional regulation driving the gene expression pattern in fibrocytes. **Tab 4:** Prediction of upstream receptors driving the gene expression pattern in fibrocytes. **Tab 5:** Fibrocyte-specific gene expression data used to formulate functional gene networks.

Available for download at
https://journals.biologists.com/bio/article-lookup/doi/10.1242/bio.062486#supplementary-data

Dataset 2. Transcriptomic effects driven by fibrocyte medium (FbC) conditions. Tab 1: Genes differentially expressed between fibrocytes in FbC vs Fb medium conditions. **Tab 2:** Canonical pathways enriched in fibrocytes in FbC medium compared to fibrocytes in Fb medium. **Tab 3:** Genes regulated by FbC medium factors: dexamethasone, WNT signaling, TGFB1 inhibition, and EGF signaling.

Available for download at
https://journals.biologists.com/bio/article-lookup/doi/10.1242/bio.062486#supplementary-data

Biology Open • Supplementary information

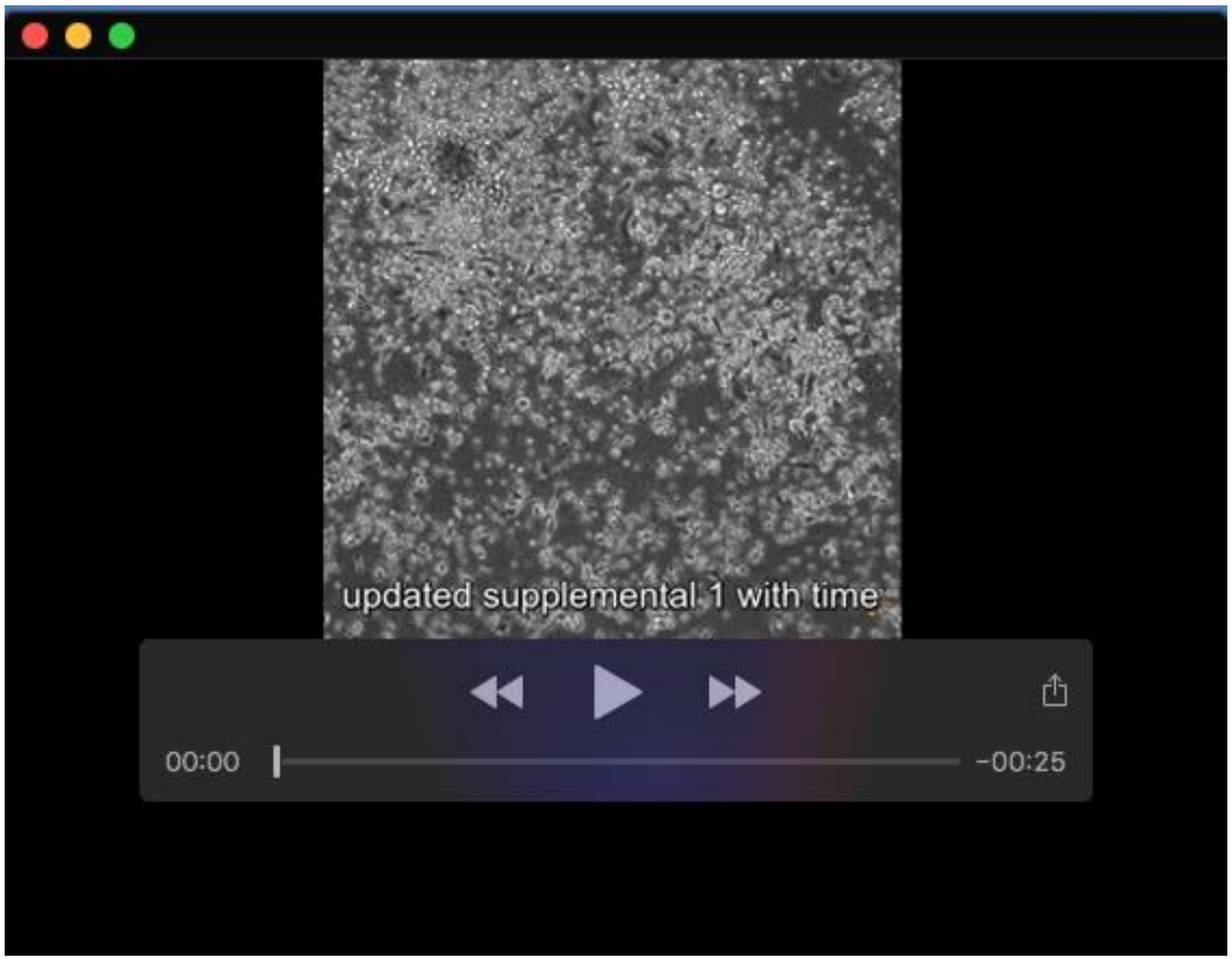

Movie 1. Fibrocyte outgrowths from peripheral blood-derived cells. Time-lapse phase-contrast imaging of adherent peripheral blood-derived cells proliferating from a nucleation site on a gelatin-coated culture plate maintained in FbC medium. During the first 48 hours, numerous non-adherent cells and debris are present while attachment occurs. The sequence begins 24 hours after plating, showing the initial appearance of small adherent cell populations. A wash and medium change, indicated in the video, removes most non-adherent material. In this field, a nucleation site resembling a colony-forming unit becomes evident, from which fibrocytes proliferate outward and actively migrate across the plate, while the central region remains densely populated due to sustained proliferation. Images were acquired every 10 minutes and compiled at 7 frames per second, with the lower-right time stamp indicating elapsed minutes. Scale bar = 10 μm.

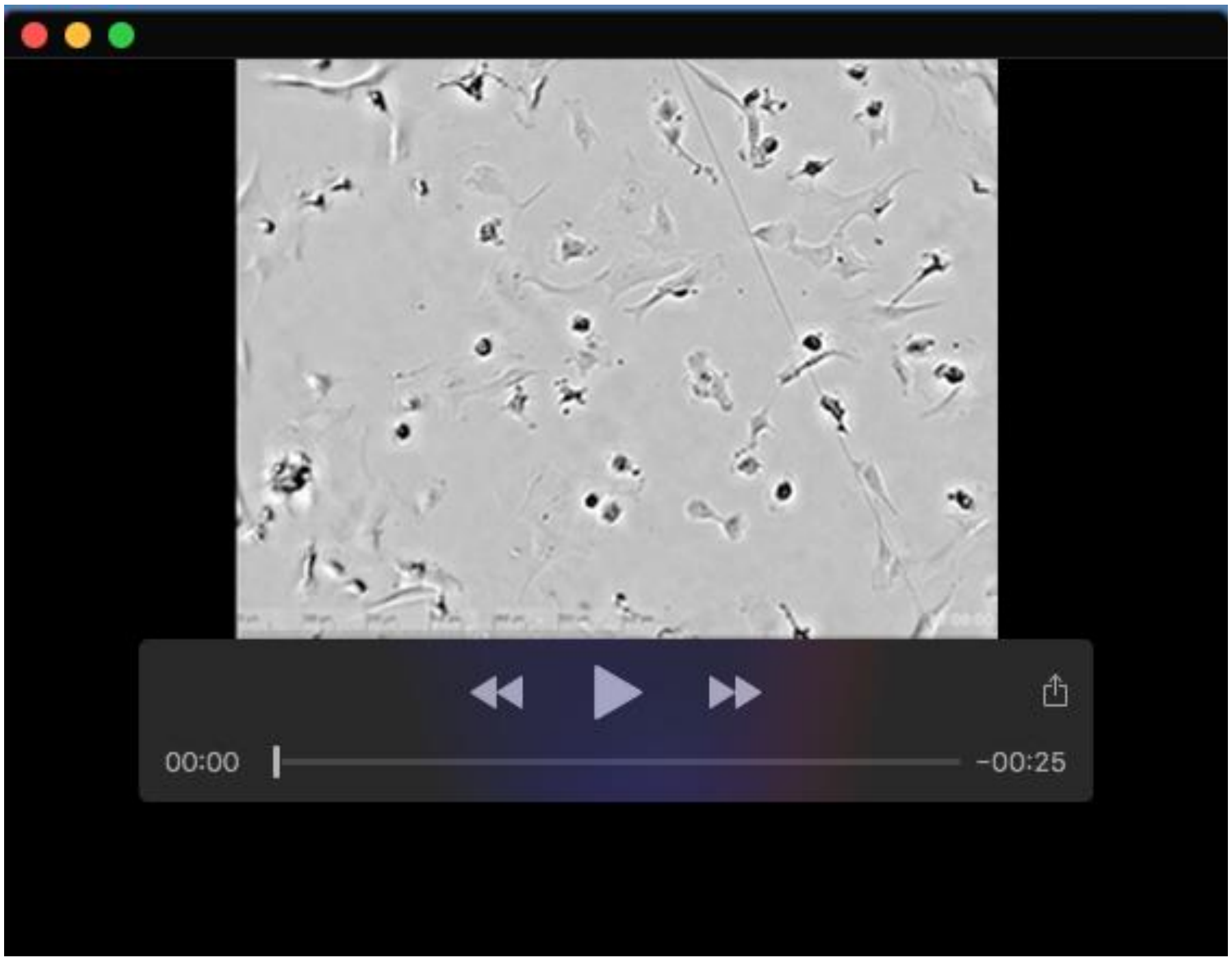

Movie 2. Fibrocyte proliferation in Fb medium. Time-lapse phase-contrast imaging showing fibrocyte proliferation in Fb medium. Fibrocytes previously maintained in Fb medium for approximately 10 days were passaged once and seeded at 3.0 x 10^4 cells per well in a 24-well plate. The video captures cell migration, progressive attachment, elongation, and proliferation of fibrocyte-derived cells, with frames highlighting morphological transitions during cell division. The population shows a diffuse growth pattern with spindle-shaped cells displaying broadened lamellipodia and thick filopodia. Over time, extensive migratory activity and morphological changes becomes evident, with formation of polygonal cells and with some cells enriched in stress fibers. Images were acquired every 10 minutes for 7 days and compiled at 5 frames per second. Scale bar = 600 μm.

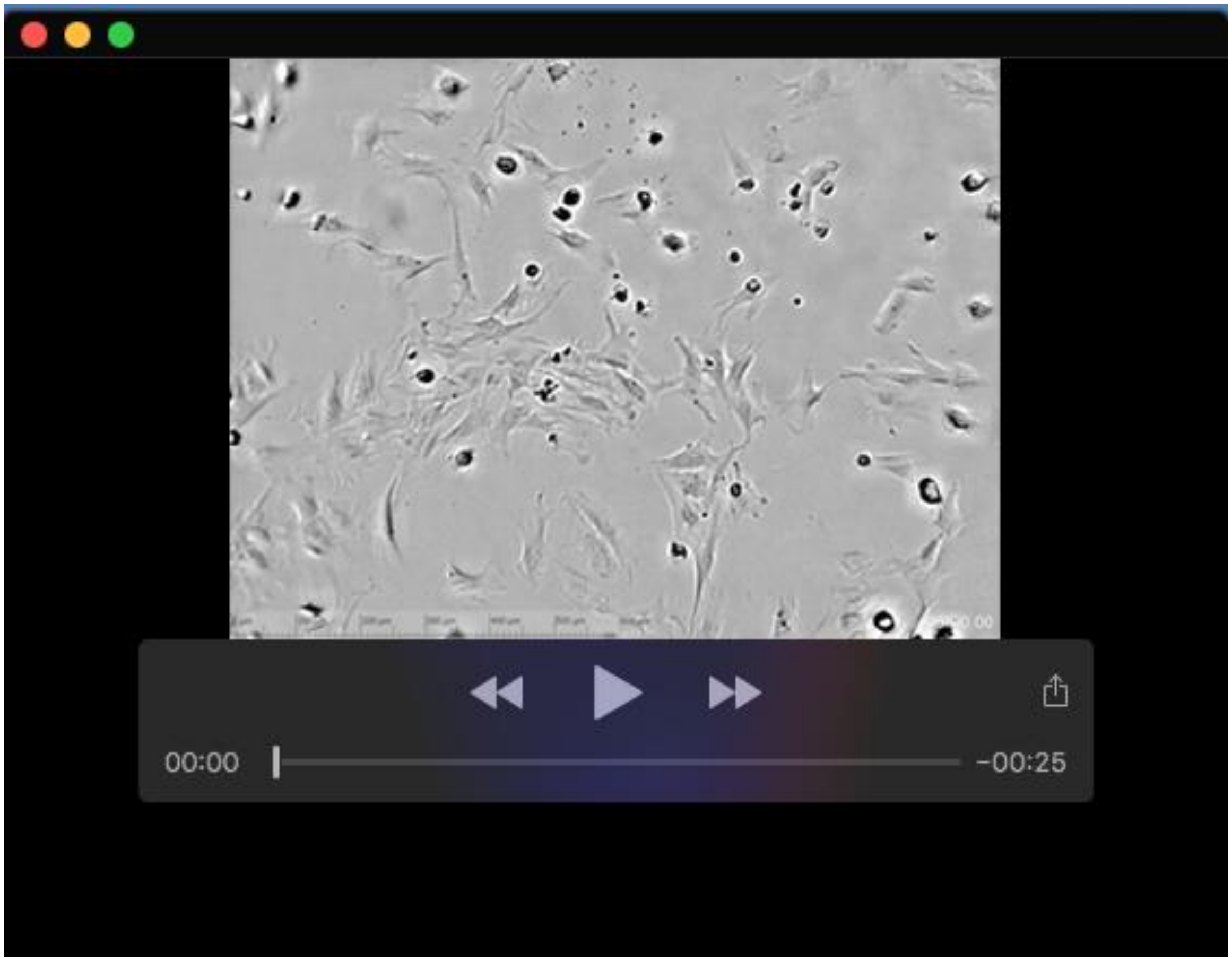

Movie 3. Fibrocyte proliferation in FbC medium. Time-lapse phase contrast imaging showing fibrocyte proliferation in FbC medium. Fibrocytes previously maintained in FbC medium for approximately 10 days were passaged once and seeded at 3.0 x 10^4 cells per well in a 24-well plate. The video shows gradual cell attachment and spreading, with specific frames highlighting distinct mitotic events. The culture is composed of fusiform, squamous, and pyramidal-shaped cells exhibiting organized palisading projections. Compared to Fb medium, cells display reduced migratory behavior and enhanced proliferative activity. Images were acquired every 10 minutes over 7 days and compiled at 5 frames per second. Scale bar = 600 µm.
