# Supplementary material for: Signal-integrated control of immune and reparative programs establishes blood-derived fibrocytes reprogrammable toward induced pluripotency
Source: Biol Open. 2026 Sep 29;15(9):bio062486. doi: 10.1242/bio.062486 (PMC13670359; doi:10.1242/bio.062486)
Supplement: Supplementary file 2 [file biolopen-15-062486_review_history.pdf]

Signal-integrated control of immune and reparative programs establishes blood-derived fibrocytes reprogrammable toward induced pluripotency

Hannah Sylvester, Prasanthi P. Koganti, Shailesh Gurung, Viju V. Pillai and Vimal Selvaraj
10.1242/bio.062486

Editor: Alissa Armstrong

Review timeline

Original submission:	12 January 2026
Editorial decision:	20 January 2026
First revision received:	18 May 2026
Editorial decision:	28 May 2026
Second revision received:	21 August 2026
Accepted:	25 August 2026

Original submission

First decision letter

MS ID#: bio.062486

MS Title: Signal-integrated control of immune and reparative programs establishes blood-derived fibrocytes reprogrammable toward induced pluripotency

Authors: Hannah Sylvester; Prasanthi P. Koganti; Shailesh Gurung; Viju V. Pillai; Vimal Selvaraj

I have now reached a decision on the above manuscript.

The reviewer reports are shown at the bottom of this email.

As you will see, the reviewers raised a number of substantial criticisms that prevent me from accepting the paper at this stage.

They suggest, however, that a revised version might prove acceptable, if you can address their concerns. If you think that you can deal satisfactorily with the criticisms on revision, I would be pleased to see a revised manuscript. We would then return it to the reviewers.

At this stage, we also ask you to ensure your manuscript complies with our formatting guidelines. Provided you are able to fully address the referees' comments, we are positive about publication of your paper (we accept over 95% of revision submissions) and therefore hope you won't mind any extra work involved in reformatting your manuscript at this point.
Please upload both a 'clean' version of your Word file, along with a highlighted version clearly showing where you have made changes in the revised manuscript. Please avoid using 'Track changes' in Word files as these are lost in PDF conversion.

I should be grateful if you would also provide a point-by-point response detailing how you have dealt with the points raised by the reviewers in the 'Response to Reviewers' box. Please attend to

© 2026. Published by The Company of Biologists under the terms of the Creative Commons Attribution License (https://creativecommons.org/licenses/by/4.0/).

all of the reviewers' comments. If you do not agree with any of their criticisms or suggestions please explain clearly why this is so.

Reviewer 1

Comments for the author

In this manuscript, the authors seek to address two points: they isolate and molecularly characterise fibrocytes from cattle; and they expand these cells as somatic starting material for reprogramming to induced pluripotent stem cells (iPSCs).

I find that both aspects are somewhat underdeveloped and would warrant additional work to solidify the conclusions made in the paper and presentation of the data.

I find it hard to pin-point what the main aspect of the paper is. If the main focus is on the molecular characterisation of bovine fibrocytes, a comparison to fibrocytes from other species is missing. If the focus is on the reprogramming of these cells, a comparison of their reprogramming efficiency with e.g. bovine fibroblasts is missing. I do recognise the authors discuss how the sample collection for fibrocytes is significantly easier as compared to fibroblasts. This aspect is not sufficiently emphasised in the discussion, and I would encourage the authors to expand on their views why this is particularly useful.

The authors also invest the time of developing an improved media condition. The potential to improve the culture of fibrocytes from other species is sadly (but understandably) not explored in this manuscript, but should certainly be discussed, to project future direction.

Additionally, a lot of the characterisation of the derived cells as 'fibrocytes' rests on their morphological description. As someone unfamiliar with this cell type and the field, I find it difficult to identify the key morphological characteristics in the data provided. Contrasting with bovine fibroblasts and comparison with fibrocytes from other species would be tremendously beneficial.

Here is my more detailed reasoning, guided along the questions prompted by the Open Review process.

Do all the figures have the proper controls?
No. Figure 7 shows a Western Blot without loading control. Preferably the authors should show the equivalent Western Blot on fibrocytes, which should show the obvious absence of these pluripotency-associated genes. The authors could also show the loss of fibrocyte-associated genes in reprogrammed cells.

The transcriptional comparison of fibrocytes and fibroblasts used fibroblasts not derived in this study or by this group. Preferable would be a fibroblast control from the same animal, derived in parallel. This limitation is not discussed sufficiently.

Were the data analysed using appropriate statistical tests?
No. Figure 3 used statistical analysis, without specifying which test was used. This information is not listed in the methods section either. Other figures show 'representative' images, but the sample size is not stated (e.g. Fig 1, 3, and 7). This should be checked for all figures.

Were experiments performed using adequate number of biological replicates?

No. As stated above, in multiple instances the sample size is not explicitly stated. For the computational analyses it's unclear how many replicates were analysed.

The authors do not disclose the efficiency of their reprogramming, although making references to the cell type being particularly suitable for reprogramming. This analysis is crucial to strengthen a key selling point of their work and would greatly elevate it.

Does the methods section provide sufficient detail to permit reproducibility?

© 2026. Published by The Company of Biologists under the terms of the Creative Commons Attribution License (https://creativecommons.org/licenses/by/4.0/).

No. The computational analysis underlying Figure 6 is unclear to me.

Are the manuscript's conclusions supported by the data?

I selected no, because it could be better.

The authors conclude that the cell type they derive from blood are fibrocytes, based on the cell morphology. The authors also describe morphological changes to the cells during derivations. To the untrained eye, these are not immediately accessible in the images and videos provided. Please consider marking specific examples of the cell morphology. Could the authors please compare the morphology of bovine fibrocytes to fibroblasts, and preferably fibrocytes of other species, if there is a shared appearance.

The authors also state that cell morphology was maintained over passages, but only show two passages, while a longer comparison would be required to make that statement.

The authors show a transcriptional difference between fibrocytes and fibroblasts, but not a similarity with established fibrocyte cell lines from other species.

The authors emphasise the incomplete molecular characterisation of fibrocytes. However, they list several genes, which characterise fibrocytes, based on other work, but don't highlight the expression of these genes in their data set. Making these selected genes more visible, would greatly help convince the reader that fibrocytes were derived.

Regarding the molecular analysis of fibrocytes, the authors make assumptions regarding the cells' function in their adapted fibrocyte medium, which are not supported by functional experiments. Without functional experiments, this should be discussed as indication for future direction.

The authors developed an improved culture condition for the expansion of fibrocytes. How the authors arrived at the media composition is not apparent or discussed. This reasoning would help to make the much more complex media conditions easily acceptable to the community.
In Figure 5, the contribution of the individual signalling modulators to the transcriptional change is described, based on association of molecules with pathways they modulate and differential gene expression.
How the authors arrive at the conclusion of which genes are regulated by which signalling molecule is not sufficiently discussed.

Regarding the reprogramming experiment, the authors show some data supporting that the cells acquire a pluripotent cell state. However, the Western Blot showing pluripotency-associated gene expression is incomplete. The control of fibrocytes is missing. The ability of the reprogrammed cells to give rise to cell types of all three germ layers has not been demonstrated, although the lab has published teratoma-formation assays as recently as 2021. Alternatively, the authors could perform transcriptional comparison of their reprogrammed cell lines with other bovine iPSC cell lines derived in their own lab to confirm their similarity.

As mentioned above, the reprogramming efficiency is not indicated, which would be important to judge the utility of this source of somatic cells for reprogramming.

It is unclear to me whether stable cell lines were established, which again is crucial to demonstrate the utility of fibrocytes as starting material in reprogramming.

Did the authors cite and discuss the merits of data that would argue for and against their conclusions?

My understanding is that bovine fibrocytes had not previously been derived or reprogrammed. Therefore, there is limited comparison to be made.

© 2026. Published by The Company of Biologists under the terms of the Creative Commons Attribution License (https://creativecommons.org/licenses/by/4.0/).

However, the authors could compare the transcriptome of bovine fibrocytes to those of other species, which seem to exist in mouse and human (doi: 10.1002/stem.1648, doi: 10.3389/fcvm.2021.753711).

They definitely should compare the efficiency of their reprogramming of bovine fibrocytes to bovine fibroblast reprogramming efficiency, which is missing in this manuscript, but would strengthen their point. I recognise the authors do speculate about this in the discussion, and reprogramming is time-consuming and expensive. I therefore suggest the authors compare their fibrocyte reprogramming efficiencies with the efficiencies recorded for their lab's publication in 2021 (https://doi.org/10.1242/bio.058756) to minimize additional work needed.

Regarding the writing style of the manuscript, I would recommend to incorporate the 2nd and 3rd paragraph of the discussion (p12 line 24 to p14 line 29) into the introduction. Additionally, some parts of the discussion sound more like a justification of the methods used in the study. I would encourage the authors to compare and contrast their findings with previous work, to provide a bigger picture on the novel understanding and opportunity gained through their work.

With some adjustments, I can see merit in publishing this manuscript.

Reviewer 2

Comments for the author

In this manuscript, the authors describe the derivation and molecular characterization of fibrocytes from adult bovine peripheral blood for the first time. They establish a reproducible protocol for fibrocyte isolation and expansion under conditions compatible with field collection, and define the transcriptional identity of these cells using RNA sequencing and network-based analyses. Comparative analyses demonstrate that bovine fibrocytes are molecularly distinct from fibroblasts, exhibiting enrichment of immune, inflammatory, metabolic, and stress-responsive pathways alongside mesenchymal features. The authors further develop an optimized fibrocyte culture medium that promotes stable proliferation and mesenchymal cytoskeletal and matrix-related programs while reducing inflammatory signaling. Finally, they show that bovine fibrocytes can be reprogrammed using polycistronic OCT4-SOX2-KLF4-cMYC and SV40 large T antigen to generate induced pluripotent stem cell colonies with characteristic molecular and morphological features of pluripotency.

I have a few comments and recommendations that I believe would further strengthen the manuscript.

1. The authors use RNA-seq-based analyses (differential gene expression, PCA clustering, upstream regulator inference, and receptor-level pathway enrichment) to verify fibrocyte identity in cells isolated from bovine serum. While these analyses clearly demonstrate that the isolated population is transcriptionally distinct from fibroblasts, transcriptomic differences alone are insufficient to conclusively establish fibrocyte identity. Fibrocytes are classically defined by the co-expression of hematopoietic and mesenchymal markers at the protein level (e.g., CD45 and collagen I), and pathway enrichment or inferred upstream regulators cannot substitute for such validation.

To substantiate the identity of bovine fibrocytes and meet the standards of experimental rigor expected for cell-type definition, the authors should include at least one orthogonal, protein-level validation experiment. Specifically, either (i) flow cytometric demonstration of $CD45^{+}$ collagen I^{+} cells using bovine-reactive antibodies, or (ii) immunofluorescence demonstrating co-localization of CD45 (or another leukocyte marker) and collagen I within the same cell, would be sufficient.

2. The authors state that DESeq2 was used for count normalization, while differential expression testing was performed using edgeR's quasi-likelihood framework. As edgeR typically applies its own normalization, it would be helpful for clarity and reproducibility if the authors could briefly justify this choice and clarify how normalized counts were incorporated into the edgeR analysis.

© 2026. Published by The Company of Biologists under the terms of the Creative Commons Attribution License (https://creativecommons.org/licenses/by/4.0/).

3. The authors analyzed ALP activity and transcription of bovine pluripotency genes (OCT4, SOX2, KLF4 etc). While these are appropriate markers for bovine iPSCs, additional validation by protein-level detection (e.g., OCT4, SOX2, NANOG (Intracellular markers) and SSEA-4 (surface marker) immunostaining) is required.

4. Functional differentiation assays to demonstrate that fibrocyte-derived biPSCs can generate derivatives of all three germ layers is required. This would strengthen the evidence that fibrocyte-derived biPSCs are truly pluripotent. You can refer to the paper: https://pubmed.ncbi.nlm.nih.gov/28720304/

5. The discussion section is quite lengthy. It could be more structured by adding key points such as comparison with existing literature, explicit limitations, and actionable future research directions. I suggest condensing redundant parts and incorporating these elements to enhance clarity and impact.

6. The study provides a valuable method to derive and expand blood-derived fibrocytes, characterizes them transcriptomically, and demonstrates their reprogrammability into iPSCs. However, the current title emphasizes immune and reparative programs but does not clearly convey the iPSC outcome. I suggest including the iPSC reprogramming aspect in the title.

7. Scale bars are not depicted in all microscopic images. Please add them for consistency and clarity.

8. The legends currently provide extensive explanations of the results. It would improve clarity if the figure legends are concise, providing only essential information to interpret the figures.

9. Figure 5 shows panel B, but panel A is not included or labelled. Please ensure all figure panels are correctly labelled and presented.

Reviewer's Responses to Questions

Experimental quality
Does each figure have the proper controls?
If 'No', please indicate reasons in Comments for Author box below.
Reviewer #1:

- No

Reviewer #2:

- Yes

Were the data analyzed using appropriate statistical tests?
If 'No', please indicate reasons in Comments for Author box below.
Reviewer #1:

- No

Reviewer #2:

- Yes

Reproducibility
Were experiments performed using adequate number of biological replicates?
If 'No', please indicate reasons in Comments for Author box below.
Reviewer #1:

- No

Reviewer #2:

- Yes

© 2026. Published by The Company of Biologists under the terms of the Creative Commons Attribution License (https://creativecommons.org/licenses/by/4.0/).

Does the methods section provide sufficient detail to permit reproducibility?
If 'No', please indicate reasons in Comments for Author box below.
Reviewer #1:

- No

Reviewer #2:

- Yes

Completeness
Are the manuscript's conclusions supported by the data?
If 'No', please indicate reasons in Comments for Author box below.
Reviewer #1:

- No

Reviewer #2:

- No

Scholarship
Do the authors cite and discuss the merits of data that would argue for and against their conclusion?
If 'No', please indicate reasons in Comments for Author box below.
Reviewer #1:

- No

Reviewer #2:

- Yes

Does the manuscript title & abstract accurately reflect the contents of the manuscript, without hyperbole?
If 'No', please indicate reasons in Comments for Author box below.
Reviewer #1:

- Yes

Reviewer #2:

- No

First revision

Author response to reviewers' comments

Response to reviewer comments (in blue)

Editor comments:

As you will see, the reviewers raised a number of substantial criticisms that prevent me from accepting the paper at this stage.

They suggest, however, that a revised version might prove acceptable, if you can address their concerns. If you think that you can deal satisfactorily with the criticisms on revision, I would be pleased to see a revised manuscript. We would then return it to the reviewers.

At this stage, we also ask you to ensure your manuscript complies with our formatting guidelines.

© 2026. Published by The Company of Biologists under the terms of the Creative Commons Attribution License (https://creativecommons.org/licenses/by/4.0/).

Provided you are able to fully address the referees' comments, we are positive about publication of your paper (we accept over 95% of revision submissions) and therefore hope you won't mind any extra work involved in reformatting your manuscript at this point.

We thank you for your constructive assessment of our manuscript and for the opportunity to submit a revised version.

We also appreciate that, despite raising points of concern, both reviewers recognized the merit of the study. The manuscript introduces a previously unexplored bovine blood- derived cell source, establishes conditions for its expansion, defines its molecular distinction from conventional fibroblasts, and demonstrates its potential use as a somatic substrate for iPSC generation. These elements together form the central contribution of the study: positioning bovine fibrocytes as a practical and field-accessible platform for reprogramming and future livestock biotechnology.

We have revised the manuscript accordingly, with the goal of making the biological identity, practical utility, and broader significance of bovine fibrocytes much clearer to readers.

Comments from the Reviewers:

Reviewer 1: In this manuscript, the authors seek to address two points: they isolate and molecularly characterise fibrocytes from cattle; and they expand these cells as somatic starting material for reprogramming to induced pluripotent stem cells (iPSCs).

I find that both aspects are somewhat underdeveloped and would warrant additional work to solidify the conclusions made in the paper and presentation of the data.

We thank the reviewer for recognizing the two major experimental advances presented in the manuscript: derivation and molecular characterization of bovine fibrocytes, and demonstration that these cells can serve as a somatic substrate for iPSC generation. We respectfully clarify that the manuscript was not intended to be either a comprehensive cross-species fibrocyte biology study or a full mechanistic study of pluripotency acquisition. Rather, the central advance is the establishment of bovine peripheral blood- derived fibrocytes as a practical, expandable, and reprogrammable somatic cell platform. These elements are necessarily linked: without defining and expanding the fibrocyte population, its utility for reprogramming cannot be evaluated; conversely, the biological and practical significance of the cell type is strengthened by demonstrating its use in iPSC generation.

We agree that this framing could be made clearer. We have therefore revised parts of the Introduction and Discussion to state these points more explicitly.

I find it hard to pin-point what the main aspect of the paper is. If the main focus is on the molecular characterisation of bovine fibrocytes, a comparison to fibrocytes from other species is missing. If the focus is on the reprogramming of these cells, a comparison of their reprogramming efficiency with e.g. bovine fibroblasts is missing.

We appreciate this point and have revised the manuscript to make the central focus more explicit. The manuscript is best understood as a platform paper: it introduces bovine fibrocytes as a previously underutilized blood-derived somatic cell source, establishes conditions for their expansion, defines their molecular distinction from fibroblasts, and demonstrates their use for reprogramming. The purpose is therefore not solely fibrocyte taxonomy, nor solely reprogramming efficiency, but the integration of these components into a practical bovine biotechnology workflow.

We agree that comparisons to fibrocytes from other species and to bovine fibroblast reprogramming are useful contextual benchmarks. However, we respectfully note that neither comparison is a prerequisite for establishing the central advance of the study.

Bovine fibrocytes have not previously been developed as a reprogramming substrate, and the ability to isolate, expand, molecularly profile, and reprogram these cells already establishes a new

© 2026. Published by The Company of Biologists under the terms of the Creative Commons Attribution License (https://creativecommons.org/licenses/by/4.0/).

practical entry point for bovine iPSC generation.

I do recognise the authors discuss how the sample collection for fibrocytes is significantly easier as compared to fibroblasts. This aspect is not sufficiently emphasised in the discussion, and I would encourage the authors to expand on their views why this is particularly useful.

We agree with the reviewer that this practical point is central to the value of the study and have indicated this in the Discussion accordingly. In cattle, somatic cell access is not a trivial issue. Skin biopsies require restraint, local preparation, tissue collection, wound care, and greater logistical coordination, whereas peripheral blood collection is routine, minimally invasive, repeatable, compatible with field conditions, and independent of age, sex, and pregnancy status. For genetic preservation, livestock biobanking, and field-based sampling of elite or geographically dispersed animals, this distinction is highly consequential.

The authors also invest the time of developing an improved media condition. The potential to improve the culture of fibrocytes from other species is sadly (but understandably) not explored in this manuscript, but should certainly be discussed, to project future direction.

We have been careful not to overstate the cross-species applicability of the medium because this was not tested experimentally. However, the development of an improved fibrocyte expansion condition in cattle may have broader relevance because fibrocyte culture remains technically challenging in multiple species. We have now included a statement in the discussion indicating this as an important future development.

Additionally, a lot of the characterisation of the derived cells as 'fibrocytes' rests on their morphological description. As someone unfamiliar with this cell type and the field, I find it difficult to identify the key morphological characteristics in the data provided. Contrasting with bovine fibroblasts and comparison with fibrocytes from other species would be tremendously benefitial.

We respectfully clarify that our identification of the cells as fibrocytes does not rest solely on morphology. The manuscript combines peripheral blood origin, culture behavior, fibrocyte-like morphology, mesenchymal features, and transcriptomic distinction from conventional fibroblasts. Morphology is therefore one part of a broader identity framework to be comprehensive, not the sole basis for classification.

Here is my more detailed reasoning, guided along the questions prompted by the Open Review process.

Do all the figures have the proper controls?

No. Figure 7 shows a Western Blot without loading control. Preferably the authors should show the equivalent Western Blot on fibrocytes, which should show the obvious absence of these pluripotency-associated genes. The authors could also show the loss of fibrocyte- associated genes in reprogrammed cells.

We thank the reviewer for raising this point, but we believe there may be a misunderstanding regarding the nature of the experiment. Figure 7 is not a Western blot; it is an RT-PCR analysis of endogenous pluripotency-associated gene expression in fibrocyte- derived reprogrammed colonies. Therefore, the concern about the absence of a Western blot loading control does not apply to this panel.

The RT-PCR was included as a validation assay to show that the resultant colonies express a panel of genes widely used to assess pluripotency and reprogramming, including POU5F1/OCT4, LIN28, NANOG, SOX2, ESRRB, SALL4, and STELLA/DPPA3. The goal was not to perform a comparative analysis between primary fibrocytes and reprogrammed cells, nor to study the reprogramming trajectory. Rather, the purpose was to confirm that fibrocyte-derived colonies acquired endogenous

© 2026. Published by The Company of Biologists under the terms of the Creative Commons Attribution License (https://creativecommons.org/licenses/by/4.0/).

expression of established pluripotency- associated transcripts.

We also note that the starting fibrocyte population has already been characterized by RNA- seq, and the full dataset has been deposited in NCBI-GEO (GSE314454). In that dataset, the pluripotency-associated genes assessed in Figure 7 are not expressed in fibrocytes. Thus, the transcriptomic data already provide the relevant starting-cell context. We have included this point in the Results section of the revised manuscript.

The transcriptional comparison of fibrocytes and fibroblasts used fibroblasts not derived in this study or by this group. Preferable would be a fibroblast control from the same animal, derived in parallel. This limitation is not discussed sufficiently.

We thank the reviewer for this comment. We respectfully note that the concept of a single “fibroblast control” is itself complicated, because fibroblasts are highly tissue- and context-dependent (Dermal fibroblasts, lung fibroblasts, uterine stromal fibroblasts, tendon fibroblasts, adventitial fibroblasts, embryonic fibroblasts, etc. all differ transcriptionally). A same-animal fibroblast control would still require choosing a particular tissue source, and that choice would influence the transcriptomic comparison – beyond a specific subset of common transcriptional identity. Thus, such a control would not necessarily represent a more appropriate fibroblast baseline; it would just represent one additional tissue-derived fibroblast population.

Our objective was to use the fibroblast transcriptional identity as a reference comparator to ask whether the blood-derived cells established here have distinguishable features (particularly targeting features that might reveal their immune cell identity). The analysis supports that conclusion.

Were the data analysed using appropriate statistical tests?

No. Figure 3 used statistical analysis, without specifying which test was used. This information is not listed in the methods section either. Other figures show 'representative' images, but the sample size is not stated (e.g. Fig 1, 3, and 7). This should be checked for all figures.

We thank the reviewer for pointing out the need for clearer statistical and replicate reporting. We agree that the statistical test used for the Figure 3C comparisons was not explicitly stated in the figure legend or Methods, and we have corrected this in the revised manuscript.

Were experiments performed using adequate number of biological replicates?

No. As stated above, in multiple instances the sample size is not explicitly stated. For the computational analyses it's unclear how many replicates were analysed.

The authors do not disclose the efficiency of their reprogramming, although making references to the cell type being particularly suitable for reprogramming. This analysis is crucial to strengthen a key selling point of their work and would greatly elevate it.

We thank the reviewer for this comment and have revised the manuscript to provide clearer sample-size and replicate information throughout. For representative images and computational analyses, we now explicitly state the number of independent samples, cultures, or experiments used, as appropriate.

Regarding reprogramming efficiency, we respectfully clarify that the reprogramming experiment was included as a proof-of-principle validation of utility, not as a quantitative study of reprogramming efficiency. The central goal of the manuscript is to establish that bovine peripheral blood-derived fibrocytes can be isolated, expanded, molecularly characterized, and used as a somatic starting cell source for reprogramming. The iPSC experiment demonstrates that fibrocytes are permissive to reprogramming and can give rise to colonies expressing pluripotency-associated markers.

© 2026. Published by The Company of Biologists under the terms of the Creative Commons Attribution License (https://creativecommons.org/licenses/by/4.0/).

Does the methods section provide sufficient detail to permit reproducibility?

No. The computational analysis underlying Figure 6 is unclear to me.

We appreciate that the connection between this Methods description and Figure 6 could have been made clearer for readers. In the revised manuscript, we have added explicit cross-referencing in the Methods and revised the Figure 6 legend.

Are the manuscript's conclusions supported by the data?

I selected no, because it could be better.

The authors conclude that the cell type they derive from blood are fibrocytes, based on the cell morphology. The authors also describe morphological changes to the cells during derivations. To the untrained eye, these are not immediately accessible in the images and videos provided. Please consider marking specific examples of the cell morphology. Could the authors please compare the morphology of bovine fibrocytes to fibroblasts, and preferably fibrocytes of other species, if there is a shared appearance.

We thank the reviewer for this comment, but we respectfully disagree with the characterization that our conclusion rests on morphology alone. The manuscript does describe the morphology of the blood-derived adherent cells, because morphology is historically part of fibrocyte recognition in culture. However, our identification of these cells as fibrocytes is based on an integrated set of observations, including peripheral blood origin, adherent mesenchymal-like growth, transcriptomic distinction from fibroblasts, and enrichment of immune, inflammatory, matrix-associated, metabolic, and stress-responsive programs consistent with fibrocyte biology.

The authors also state that cell morphology was maintained over passages, but only show two passages, while a longer comparison would be required to make that statement.

We thank the reviewer for this comment. We agree that any statement regarding maintenance of morphology should be tied to the passages and culture period documented in the manuscript. Our intent was not to imply indefinite morphological stability over extended passaging, but to indicate that the characteristic fibrocyte morphology was retained during the culture and passage window used for the experiments in this study. We have revised the wording to make this more precise.

The authors show a transcriptional difference between fibrocytes and fibroblasts, but not a similarity with established fibrocyte cell lines from other species.

The study is focused on bovine fibrocytes, and to our knowledge there is no established bovine fibrocyte reference or dataset against which these cells can be directly benchmarked. Our conclusion is not that bovine fibrocytes are identical or different from fibrocytes from another species. Rather, this study establishes that fibrocytes can be reproducibly derived from bovine peripheral blood, expanded under defined culture conditions, distinguished molecularly from fibroblasts, and used as a practical blood- derived somatic cell source for iPSC generation.

The authors emphasise the incomplete molecular characterisation of fibrocytes. However, they list several genes, which characterise fibrocytes, based on other work, but don't highlight the expression of these genes in their data set. Making these selected genes more visible, would greatly help convince the reader that fibrocytes were derived.

We thank the reviewer for this suggestion. We respectfully note that the genes referenced in this section are not being used as a diagnostic marker panel to establish fibrocyte identity, nor are we making quantitative cross-study comparisons of their expression levels. They are discussed to place

© 2026. Published by The Company of Biologists under the terms of the Creative Commons Attribution License (https://creativecommons.org/licenses/by/4.0/).

the bovine fibrocyte transcriptome in the context of prior fibrocyte literature and to show that the overall transcriptional profile is consistent with known fibrocyte-associated biology.

For that reason, we do not think that elevating a small set of literature-derived genes into a central figure or primary criterion would strengthen the manuscript. Fibrocyte marker expression varies substantially across species, isolation methods, culture conditions, activation states, and analytical platforms. Presenting selected genes too prominently could incorrectly imply that fibrocytes are defined by a fixed marker set, when the strength of our study is the broader transcriptomic characterization of bovine fibrocytes as a blood- derived cell population with integrated immune, inflammatory, matrix-associated, and mesenchymal programs.

Regarding the molecular analysis of fibrocytes, the authors make assumptions regarding the cells' function in their adapted fibrocyte medium, which are not supported by functional experiments. Without functional experiments, this should be discussed as indication for future direction.

We thank the reviewer for this comment. We respectfully clarify that the molecular analyses were not intended to serve as direct functional assays of fibrocyte behavior. Rather, as is standard for transcriptomic and pathway-based analyses, these data were used to provide a predictive understanding of the cellular programs associated with fibrocyte culture and expansion under the medium condition.

The authors developed an improved culture condition for the expansion of fibrocytes. How the authors arrived at the media composition is not apparent or discussed. This reasoning would help to make the much more complex media conditions easily acceptable to the community.

In Figure 5, the contribution of the individual signalling modulators to the transcriptional change is described, based on association of molecules with pathways they modulate and differential gene expression.

How the authors arrive at the conclusion of which genes are regulated by which signalling molecule is not sufficiently discussed.

We thank the reviewer for this comment. The FbC medium was developed empirically to improve fibrocyte expansion while retaining the fibrocyte/mesenchymal phenotype. The added components were selected based on known roles in cell survival, proliferation, inflammatory modulation, extracellular-matrix remodeling, and mesenchymal cell growth. We agree that the rationale was not sufficiently explained in the submitted manuscript, and we have revised the Methods/Results to make clear why these components were tested and how the final condition was selected.

Regarding Figure 5, we clarify that the analysis does not claim direct gene-by-gene regulation by each individual medium component. Rather, we used upstream- regulator/pathway analysis as a predictive transcriptomic framework to ask whether the transcriptional response observed in FbC medium was consistent with signaling inputs represented by components of the medium, including dexamethasone-, TGFB1-, EGF-, and WNT-associated programs. Genes shown in the figure are therefore grouped according to their assignment within the upstream-regulator analysis, not presented as experimentally proven direct targets of each molecule.

Regarding the reprogramming experiment, the authors show some data supporting that the cells acquire a pluripotent cell state. However, the Western Blot showing pluripotency- associated gene expression is incomplete. The control of fibrocytes is missing. The ability of the reprogrammed cells to give rise to cell types of all three germ layers has not been demonstrated, although the lab has published teratoma-formation assays as recently as 2021. Alternatively, the authors could perform transcriptional comparison of their reprogrammed cell lines with other bovine iPSC cell lines derived in their own lab to confirm their similarity.

As mentioned above, the reprogramming efficiency is not indicated, which would be important to judge the utility of this source of somatic cells for reprogramming.

© 2026. Published by The Company of Biologists under the terms of the Creative Commons Attribution License (https://creativecommons.org/licenses/by/4.0/).

It is unclear to me whether stable cell lines were established, which again is crucial to demonstrate the utility of fibrocytes as starting material in reprogramming.

We thank the reviewer for this comment. We first clarify that the panel referred to is not a Western blot, but an RT-PCR analysis of endogenous pluripotency-associated transcript expression in fibrocyte-derived reprogrammed colonies. The purpose of this experiment was to validate the resultant colonies for expression of established pluripotency- associated genes, not to study the reprogramming trajectory or fibrocyte-marker loss.

We also respectfully clarify the scope of the study. The reprogramming experiment is presented as proof of principle that bovine fibrocytes can serve as a blood-derived somatic starting cell source for iPSC generation. The manuscript is not intended to re-establish the full functional validation of the bovine iPSC platform, which our group previously reported using the same reprogramming framework, including differentiation into derivatives of all three germ layers.

Did the authors cite and discuss the merits of data that would argue for and against their conclusions?

My understanding is that bovine fibrocytes had not previously been derived or reprogrammed. Therefore, there is limited comparison to be made.

However, the authors could compare the transcriptome of bovine fibrocytes to those of other species, which seem to exist in mouse and human (doi: 10.1002/stem.1648, doi: 10.3389/fcvm.2021.753711).

They definitely should compare the efficiency of their reprogramming of bovine fibrocytes to bovine fibroblast reprogramming efficiency, which is missing in this manuscript, but would strengthen their point. I recognise the authors do speculate about this in the discussion, and reprogramming is time-consuming and expensive. I therefore suggest the authors compare their fibrocyte reprogramming efficiencies with the efficiencies recorded for their lab's publication in 2021 (https://doi.org/10.1242/bio.058756) to minimize additional work needed.

We thank the reviewer for this comment and appreciate the recognition that bovine fibrocytes have not previously been derived or reprogrammed. This point is central to the novelty of the manuscript. Because there is no established method, reference dataset, or validated bovine fibrocyte comparator, the present study necessarily provides the foundational demonstration: that fibrocyte-like cells can be reproducibly obtained from bovine peripheral blood, expanded in culture, molecularly characterized, distinguished from fibroblast reference cells, and used as a primary somatic cell source for reprogramming.

We have revised the Discussion to make this novelty and scope clearer, while also citing relevant fibrocyte literature from other species for context. We have also clarified that reprogramming is presented as proof of principle of utility, not as a comparative efficiency study.

Regarding the writing style of the manuscript, I would recommend to incorporate the 2nd and 3rd paragraph of the discussion (p12 line 24 to p14 line 29) into the introduction. Additionally, some parts of the discussion sound more like a justification of the methods used in the study. I would encourage the authors to compare and contrast their findings with previous work, to provide a bigger picture on the novel understanding and opportunity gained through their work.

With some adjustments, I can see merit in publishing this manuscript.

We thank the reviewer for this helpful suggestion. We agree that the manuscript benefits from a clearer statement of rationale and scope earlier in the paper. Rather than moving the Discussion paragraphs into the Introduction, we revised the Introduction to better define the knowledge gap addressed by the study: bovine fibrocytes have not previously been reproducibly derived, molecularly characterized, expanded under defined culture conditions, or tested as a blood-derived somatic cell source for reprogramming. We also strengthened the practical rationale for fibrocytes in livestock settings, where peripheral blood collection offers clear advantages over tissue biopsy.

© 2026. Published by The Company of Biologists under the terms of the Creative Commons Attribution License (https://creativecommons.org/licenses/by/4.0/).

We also revised the Discussion to emphasize interpretation of the findings in relation to prior fibrocyte and bovine iPSC literature. In particular, we now more clearly frame the study as a foundational bovine fibrocyte platform.

Reviewer 2: In this manuscript, the authors describe the derivation and molecular characterization of fibrocytes from adult bovine peripheral blood for the first time. They establish a reproducible protocol for fibrocyte isolation and expansion under conditions compatible with field collection, and define the transcriptional identity of these cells using RNA sequencing and network-based analyses. Comparative analyses demonstrate that bovine fibrocytes are molecularly distinct from fibroblasts, exhibiting enrichment of immune, inflammatory, metabolic, and stress-responsive pathways alongside mesenchymal features. The authors further develop an optimized fibrocyte culture medium that promotes stable proliferation and mesenchymal cytoskeletal and matrix- related programs while reducing inflammatory signaling. Finally, they show that bovine fibrocytes can be reprogrammed using polycistronic OCT4-SOX2-KLF4-cMYC and SV40 large T antigen to generate induced pluripotent stem cell colonies with characteristic molecular and morphological features of pluripotency.

We thank the reviewer for this thoughtful and constructive summary of our manuscript. We appreciate the reviewer's recognition of the central contributions of the study.

I have a few comments and recommendations that I believe would further strengthen the manuscript.

1. The authors use RNA-seq-based analyses (differential gene expression, PCA clustering, upstream regulator inference, and receptor-level pathway enrichment) to verify fibrocyte identity in cells isolated from bovine serum. While these analyses clearly demonstrate that the isolated population is transcriptionally distinct from fibroblasts, transcriptomic differences alone are insufficient to conclusively establish fibrocyte identity. Fibrocytes are classically defined by the co-expression of hematopoietic and mesenchymal markers at the protein level (e.g., CD45 and collagen I), and pathway enrichment or inferred upstream regulators cannot substitute for such validation.

To substantiate the identity of bovine fibrocytes and meet the standards of experimental rigor expected for cell-type definition, the authors should include at least one orthogonal, protein-level validation experiment. Specifically, either (i) flow cytometric demonstration of $CD45^+$ collagen I^+ cells using bovine-reactive antibodies, or (ii) immunofluorescence demonstrating co-localization of CD45 (or another leukocyte marker) and collagen I within the same cell, would be sufficient.

We thank the reviewer for this comment and agree that CD45/collagen I co-expression is a commonly used criterion – and is also seen in gene expression our dataset. However, in our study, the cells are not identified by pathway inference alone; they are supported by peripheral blood origin, adherent fibrocyte-like culture behavior, mesenchymal morphology, and expression of both hematopoietic and mesenchymal markers in the RNA- seq dataset, including CD45/PTPRC and collagen genes. Thus, the dataset already captures the canonical mixed hematopoietic-mesenchymal signature expected of fibrocytes. Because bovine fibrocytes have not previously been established and bovine- specific protein-marker standards are not yet defined, we do not view an additional single protein co-staining assay as essential for the central conclusion of this foundational study.

2. The authors state that DESeq2 was used for count normalization, while differential expression testing was performed using edgeR's quasi-likelihood framework. As edgeR typically applies its own normalization, it would be helpful for clarity and reproducibility if the authors could briefly justify this choice and clarify how normalized counts were incorporated into the edgeR analysis.

We thank the reviewer for catching this error. DESeq2 was not used in this study. The statement referring to DESeq2 normalization was included inadvertently and has been removed. We have revised the Methods section to clarify that normalization and differential expression testing were

© 2026. Published by The Company of Biologists under the terms of the Creative Commons Attribution License (https://creativecommons.org/licenses/by/4.0/).

performed within the edgeR framework.

3. The authors analyzed ALP activity and transcription of bovine pluripotency genes (OCT4, SOX2, KLF4 etc). While these are appropriate markers for bovine iPSCs, additional validation by protein-level detection (e.g., OCT4, SOX2, NANOG (Intracellular markers) and SSEA-4 (surface marker) immunostaining) is required.

We thank the reviewer for this suggestion. We agree that protein-level pluripotency marker analysis can provide additional validation of fully established iPSC lines. However, in this manuscript the reprogramming experiment is presented as proof of principle that bovine fibrocytes can serve as a blood-derived somatic starting cell source for iPSC generation, rather than as a comprehensive characterization of a new pluripotent stem cell line. The colonies were evaluated using established criteria appropriate for this scope, including iPSC-like colony morphology, ALP activity, and endogenous expression of bovine pluripotency-associated transcripts. We have revised the manuscript to make this scope clearer and to avoid overstating the reprogramming data beyond the validation presented.

4. Functional differentiation assays to demonstrate that fibrocyte-derived biPSCs can generate derivatives of all three germ layers is required. This would strengthen the evidence that fibrocyte-derived biPSCs are truly pluripotent. You can refer to the paper: https://pubmed.ncbi.nlm.nih.gov/28720304/

As stated for the previous point, the reprogramming experiment in this manuscript is presented as proof of principle that bovine fibrocytes can serve as a blood-derived somatic starting cell source for iPSC generation, rather than as a comprehensive functional validation of a newly established pluripotent stem cell line.

5. The discussion section is quite lengthy. It could be more structured by adding key points such as comparison with existing literature, explicit limitations, and actionable future research directions. I suggest condensing redundant parts and incorporating these elements to enhance clarity and impact.

We thank the reviewer for this suggestion. We have carefully re-read the Discussion and revised it to improve structure, reduce redundancy, and focus the interpretation more clearly. We made changes where appropriate while preserving the necessary context for the study's major findings.

6. The study provides a valuable method to derive and expand blood-derived fibrocytes, characterizes them transcriptomically, and demonstrates their reprogrammability into iPSCs. However, the current title emphasizes immune and reparative programs but does not clearly convey the iPSC outcome. I suggest including the iPSC reprogramming aspect in the title.

We thank the reviewer for this helpful suggestion. We agree that the title should more clearly convey the reprogramming component of the study while retaining the focus on blood-derived fibrocyte biology. We have therefore revised the title to include the iPSC reprogramming outcome and to better reflect the full scope of the manuscript.

7. Scale bars are not depicted in all microscopic images. Please add them for consistency and clarity.

We thank the reviewer for this comment. Scale bars are provided for the microscopic images in the figures. For inset panels, separate scale bars were not added because the insets correspond to the same image field/original magnification and the scale can be inferred from the parent image. We have reviewed the figures to ensure that scale bars are present and clearly visible where needed.

© 2026. Published by The Company of Biologists under the terms of the Creative Commons Attribution License (https://creativecommons.org/licenses/by/4.0/).

8. The legends currently provide extensive explanations of the results. It would improve clarity if the figure legends are concise, providing only essential information to interpret the figures.

We thank the reviewer for this suggestion. We agree that figure legends should remain clear, while still providing sufficient explanation of the findings. Because our figures integrate multiple types of data, we have chosen to retain a comprehensive legend style to ensure that the results can be understood clearly by readers.

9. Figure 5 shows panel B, but panel A is not included or labelled. Please ensure all figure panels are correctly labelled and presented.

We thank the reviewer for pointing this out. We have fixed this error in the revised manuscript.

Second decision letter

MS ID#: bio.062486R1

MS Title: Signal-integrated control of immune and reparative programs establishes blood-derived fibrocytes reprogrammable toward induced pluripotency

Authors: Hannah Sylvester; Prasanthi P. Koganti; Shailesh Gurung; Viju V. Pillai; Vimal Selvaraj

I have now reached a decision on the above resubmission.

The reviewer reports are shown at the bottom of this email.

As you will see, the reviewers were generally satisfied with the reviews made, but raised some critical points that will require amendments to your manuscript. The majority of the concerns can be addressed by changes in the text, particularly as it relates to making conclusions more moderate to match the data. I hope that you will be able to carry these out, because we would like to be able to accept your paper.

At this stage, we also ask you to ensure your manuscript complies with our formatting guidelines - please see our manuscript preparation guidelines for details. Provided you are able to fully address the referees' comments, we are positive about publication of your paper (we accept over 95% of revision submissions) and therefore hope you won't mind any extra work involved in reformatting your manuscript at this point.

Please upload both a 'clean' version of your Word file, along with a highlighted version clearly showing where you have made changes in the revised manuscript. Please avoid using 'Track changes' in Word files as these are lost in PDF conversion.

I should be grateful if you would also provide a point-by-point response detailing how you have dealt with the points raised by the reviewers in the 'Response to Reviewers' box. Please attend to all of the reviewers' comments. If you do not agree with any of their criticisms or suggestions please explain clearly why this is so.

Reviewer 1

Comments for the author

o begin with, I would like to appreciate the changes the authors have made, which improved the manuscript:

© 2026. Published by The Company of Biologists under the terms of the Creative Commons Attribution License (https://creativecommons.org/licenses/by/4.0/).

The methods section now contains a dedicated paragraph on statistics. Statistical information has been added to the figure legends and the discussion has been extensively reworded.

I recognise further changes to the manuscript, but these named above stand out to me as most effective.

One of my main concerns was the scope of the manuscript, which I believe was insufficiently stated. In reading the replies by the authors, I find their wording in the rebuttal still clearer and more convincing than in the revised manuscript.

Especially if this paper is supposed to be a platform for others to embark on the usage of blood-derived fibrocytes as somatic cell source for reprogramming, the selling points of accessibility and expansion could be made clearer.
I have therefore chosen minor revisions to encourage them to further improve the clarity of their work.

To illustrate my position, I am providing specific examples taken from the rebuttal:
This sentence would fit into both abstract or introduction with minor rewording: "Rather, the central advance is the establishment of bovine peripheral blood- derived fibrocytes as a practical, expandable, and reprogrammable somatic cell platform." This message is unfortunately not coming across from the manuscript itself in such strong clarity. Personally, I would much prefer this information in the abstract over the genes which suggest a monocyte-derived origin of fibrocytes.

Likewise the authors' comment on the practical benefits of blood over skin sample is succinct and convincing: "In cattle, somatic cell access is not a trivial issue. Skin biopsies require restraint, local preparation, tissue collection, wound care, and greater logistical coordination, whereas peripheral blood collection is routine, minimally invasive, repeatable, compatible with field conditions, and independent of age, sex, and pregnancy status."

The need for clarity also extends in to the title, which could state the aim of deriving and characterising bovine fibrocytes as an easily-accessible, expandable somatic cell source for reprogramming in much simpler terms.

Beyond this, I would like to reply to the authors' comments to reiterate a couple of outstanding issues I have with the data presentation:

I appreciate that the characterisation of bovine fibrocytes presented in this manuscript includes morphological description for historical reasons, but extends to molecular characterisation as well.

However, figure 1 and half of figure 3 focus on the morphological description of the derived cell type. I would therefore still encourage the authors to more clearly highlight the features on which their characterisation rests in their own data, and if at all possible, provide comparison to other fibrocytes or fibroblasts.

I would like to apologise for misunderstanding the data presented in figure 7 as a Western Blot.

As this paper is supposed to serve as a platform, I would still suggest to show that reprogramming is complete in Figure 7. The absence of fibrocyte markers in reprogrammed cells would serve this purpose without repeating previous work unnecessarily.

The authors also state that bulk RNAseq of the starting population can serve to indicate the absence of pluripotency genes. I agree to the extend that the RNAseq shows that fibrocytes lack pluripotency-associated gene expression. I disagree with the referral to RNAseq data as control for RT-PCR data. In my view, the controls serve to validate the robustness of the data acquired in a particular experimental context. Either RNAseq of fibrocytes is compared with RNAseq of the reprogrammed cells, to show the change in gene expression, or RT-PCR of different samples is compared to show changes in specific genes. To strengthen the data in figure 7, I would therefore include samples of the starting cell population.

© 2026. Published by The Company of Biologists under the terms of the Creative Commons Attribution License (https://creativecommons.org/licenses/by/4.0/).

Furthermore, to demonstrate the strength of fibrocyte as a source of somatic cells for reprogramming, even a rough statistical estimate of efficiency would be helpful.

Figure 7 states that 50k cells were plated for reprogramming. How many AP+ colonies were derived per experiment? How did the number of AP+ colonies vary between both biological replicates? If the aim is to provide a platform which others may use to derive stable pluripotent cell lines from blood-derived fibrocytes, I think this information will be key to indicate the extend of time and money involved in this work.

The authors also highlight the complexity of choosing "one fibroblast control". I appreciate their reasoning. In light of this complexity, I would encourage the authors to provide explicit justification for their choice of a fibroblast RNAseq dataset used for transcriptional comparison in figure 6, which is currently missing.

I raised questions regarding the documentation and explanation of the work presented in figure 6. The authors state they provided clarification in the figure legend and methods. I can only identify a minor change in the first sentence of the figure legend and would ask the authors to point out additional changes which I am missing.

The authors also highlight that fibrocyte markers can vary: "Fibrocyte marker expression varies substantially across species, isolation methods, culture conditions, activation states, and analytical platforms."
Under these circumstances especially I would encourage the authors to identify which genes are found in mouse and human fibrocytes, as well as in bovine. I agree with the authors to not overstate these as a fixed marker set, but they might still guide the comparison and grow the field.

The authors state that the components of FcB medium were chosen for known roles in various cell biological aspects. I would be in favour of including this reasoning into the manuscript, preferably with specific justifications for the signalling pathways and molecules chosen.

I appreciate that my comments are still quite extensive, but want to emphasise that, besides the RT-PCR, they can all be addressed without additional experiments.
Rephrasing, cross-referencing published fibroctye work, and further explanation can bring out the best of this data which undoubtedly was generated from hard work, so I sincerely hope the authors will invest the time.

Reviewer 2

Comments for the author

Overall, the authors have clarified the scope and interpretation of the study appropriately. Although the additional experimental validations suggested by the reviewer were not performed, the authors have provided reasonable justification for this within the context and scope of the manuscript. I would suggest the following recommended moderation of terminology and conclusions.

I appreciate the authors' detailed response and agree that the experiments done in the study supports the presence of a fibrocyte-like population. I also recognize the challenges associated with defining bovine fibrocytes and the current limitations in established bovine-specific protein markers. That said, my main concern was not the pathway analysis itself, but rather that the evidence for cell identity remains largely transcriptomic and indirect. Detection of hematopoietic and mesenchymal transcripts by RNA-seq, even when combined with morphology and culture characteristics, does not fully replace orthogonal protein-level validation, particularly when proposing a novel cell identity in a new species context.

Given the scope of the study and the authors' clarification, I do not consider additional experiments strictly necessary for publication. However, I recommend that the language throughout the manuscript be moderated where appropriate, and by acknowledging in the Discussion that definitive protein-level validation of bovine fibrocyte identity remains to be established.

© 2026. Published by The Company of Biologists under the terms of the Creative Commons Attribution License (https://creativecommons.org/licenses/by/4.0/).

I also appreciate the authors' clarification regarding the intended scope of the reprogramming experiments as proof-of-principle rather than comprehensive characterization of fully validated bovine iPSC lines. I agree that extensive pluripotency validation may be beyond the scope of the current study. However, the manuscript should consistently avoid language implying definitive establishment of fully characterized pluripotent stem cell lines unless additional protein-level or functional pluripotency assays are provided.

Thank you for the clarification regarding the scale bars. However, in Figure B the Day 7 panel appears to be an independent image rather than a magnified inset, and no scale bar is shown. Similarly, in Figure C only one of the two panels contains a scale bar. For clarity and consistency, please add scale bars to all independently displayed microscopy panels.

Reviewer's Responses to Questions

Experimental quality
Are experiments performed using appropriate methods that will answer the question (or test the hypothesis or support the observations) posed by the authors? Is the right tool used for the job?
If 'No', please indicate reasons in Comments for Author box below.
Reviewer #1:
- No

Reviewer #2:
- Yes

Were the data analyzed using appropriate statistical tests?
If 'No', please indicate reasons in Comments for Author box below.
Reviewer #1:
- Yes

Reviewer #2:
- Yes

Reproducibility
Were experiments performed using adequate number of biological replicates?
If 'No', please indicate reasons in Comments for Author box below.
Reviewer #1:
- Yes

Reviewer #2:
- Yes

Is there sufficient raw data to assess the rigor of the analysis?
If 'No', please indicate reasons in Comments for Author box below.
Reviewer #1:
- Yes

Reviewer #2:
- Yes

Does the methods section provide sufficient detail to permit reproducibility?
If 'No', please indicate reasons in Comments for Author box below.
Reviewer #1:
- No

© 2026. Published by The Company of Biologists under the terms of the Creative Commons Attribution License (https://creativecommons.org/licenses/by/4.0/).

Reviewer #2:

- Yes

Completeness
Are the manuscript's conclusions supported by the data?
If 'No', please indicate reasons in Comments for Author box below.
Reviewer #1:

- No

Reviewer #2:

- Yes

Scholarship
Do the authors cite and discuss the merits of data that would argue for and against their conclusion?
If 'No', please indicate reasons in Comments for Author box below.
Reviewer #1:

- Yes

Reviewer #2:

- No

Does the manuscript title & abstract accurately reflect the contents of the manuscript, without hyperbole?
If 'No', please indicate reasons in Comments for Author box below.
Reviewer #1:

- Yes

Reviewer #2:

- Yes

Second revision

Author response to reviewers' comments

Response to Reviewers

Reviewer 1

Comment: One of my main concerns was the scope of the manuscript, which I believe was insufficiently stated. In reading the replies by the authors, I find their wording in the rebuttal still clearer and more convincing than in the revised manuscript. Especially if this paper is supposed to be a platform for others to embark on the usage of blood-derived fibrocytes as somatic cell source for reprogramming, the selling points of accessibility and expansion could be made clearer. I have therefore chosen minor revisions to encourage them to further improve the clarity of their work.

To illustrate my position, I am providing specific examples taken from the rebuttal:

This sentence would fit into both abstract or introduction with minor rewording: "Rather, the central advance is the establishment of bovine peripheral blood- derived fibrocytes as a practical, expandable, and reprogrammable somatic cell platform." This message is unfortunately not coming across from the manuscript itself in such strong clarity. Personally, I would much prefer this

© 2026. Published by The Company of Biologists under the terms of the Creative Commons Attribution License (https://creativecommons.org/licenses/by/4.0/).

information in the abstract over the genes which suggest a monocyte-derived origin of fibrocytes.

Likewise, the authors' comment on the practical benefits of blood over skin sample is succinct and convincing: "In cattle, somatic cell access is not a trivial issue. Skin biopsies require restraint, local preparation, tissue collection, wound care, and greater logistical coordination, whereas peripheral blood collection is routine, minimally invasive, repeatable, compatible with field conditions, and independent of age, sex, and pregnancy status."

Response: We appreciate the reviewer's comment, but respectfully note that this point is already stated directly in the Abstract and Introduction. The Abstract states: "Seeking somatic cells that could be collected aseptically and reproducibly under field conditions for reprogramming to pluripotency, we recognized fibrocytes as a practical and previously unexplored candidate population." It further states: "Here, we establish a reproducible method for fibrocyte derivation and expansion from adult bovine blood and define their molecular identity using transcriptomic and network analyses." The final sentence of the Abstract also explicitly states: "These findings establish fibrocytes as a field-adaptable, stably expandable, and reprogrammable somatic cell type with practical applications in induced pluripotent stem cell generation, genetic preservation, and reproductive biotechnology."

The Introduction also already makes this practical rationale explicit: "Unlike traditional sources such as skin fibroblasts or embryonic cells, fibrocytes offer a distinct advantage as a readily accessible, diploid somatic cell population that can be isolated from peripheral blood. This enables minimally invasive collection, making fibrocytes an ideal cell source for field applications where aseptic sampling is critical." The following sentence further contrasts blood collection with biopsy-based approaches: "Compared to skin biopsies, which require specialized handling and pose a higher risk of microbial contamination, blood can be collected via simple venipuncture and either immediately processed or transported for culture under controlled conditions, allowing for the establishment of a stable cell population suitable for reprogramming."

We therefore believe the manuscript already states the platform rationale clearly.

Comment: The need for clarity also extends in to the title, which could state the aim of deriving and characterising bovine fibrocytes as an easily-accessible, expandable somatic cell source for reprogramming in much simpler terms.

Response: We appreciate the suggestion, but we have retained the current title. The title accurately reflects the biological scope of the study, which is not limited to accessibility or expansion, but also includes the signal-integrated immune and reparative programs that define the fibrocyte state and support reprogrammability. The reviewer's suggested emphasis is already captured in the Abstract and Introduction, as noted above, where we explicitly state that fibrocytes are practical, blood-derived, field- adaptable, expandable, and reprogrammable. We therefore do not believe a title change is necessary.

Comment: Beyond this, I would like to reply to the authors' comments to reiterate a couple of outstanding issues I have with the data presentation:

I appreciate that the characterisation of bovine fibrocytes presented in this manuscript includes morphological description for historical reasons, but extends to molecular characterisation as well.

However, figure 1 and half of figure 3 focus on the morphological description of the derived cell type. I would therefore still encourage the authors to more clearly highlight the features on which their characterisation rests in their own data, and if at all possible, provide comparison to other fibrocytes or fibroblasts.

Response: We appreciate the reviewer's acknowledgment that our characterization extends well beyond morphology to include molecular features/characterization. We would, however, respectfully disagree that the morphological documentation in Figures 1 and 3 is excessive. The derivation and propagation are documented clearly in the videos. To our knowledge, previous studies of fibrocytes have not provided a detailed morphological record of their emergence and maturation in culture, nor have they provided sufficiently high-quality images that could serve as a

© 2026. Published by The Company of Biologists under the terms of the Creative Commons Attribution License (https://creativecommons.org/licenses/by/4.0/).

useful reference for comparison. We considered it important to document these features carefully and provide representative images of sufficient quality to establish a visual record of the bovine fibrocyte phenotype.

Comment: As this paper is supposed to serve as a platform, I would still suggest to show that reprogramming is complete in Figure 7. The absence of fibrocyte markers in reprogrammed cells would serve this purpose without repeating previous work unnecessarily.

The authors also state that bulk RNAseq of the starting population can serve to indicate the absence of pluripotency genes. I agree to the extend that the RNAseq shows that fibrocytes lack pluripotency- associated gene expression. I disagree with the referral to RNAseq data as control for RT-PCR data. In my view, the controls serve to validate the robustness of the data acquired in a particular experimental context. Either RNAseq of fibrocytes is compared with RNAseq of the reprogrammed cells, to show the change in gene expression, or RT-PCR of different samples is compared to show changes in specific genes. To strengthen the data in figure 7, I would therefore include samples of the starting cell population.

Response: We appreciate the reviewer's perspective. However, we respectfully disagree that the starting fibrocyte population must be included as an RT-PCR control. The RNA-seq analysis comprehensively demonstrates that fibrocytes do not express pluripotency-associated genes, whereas the RT-PCR analysis addresses a different question, whether the pluripotency genes are induced following reprogramming. Thus, the two pieces of information are complementary. We do not consider it necessary to repeat, using a lower-throughput assay, information that has already been established more comprehensively by transcriptome-wide RNA-seq.

Similarly, while complete loss of fibrocyte markers would further strengthen evidence of reprogramming, we believe this extends beyond the scope of the present study. As stated in the manuscript, our objective is to provide proof-of-principle that blood-derived fibrocytes can be reprogrammed toward induced pluripotency, rather than to comprehensively characterize fully established bovine iPSC lines.

Comment: Furthermore, to demonstrate the strength of fibrocyte as a source of somatic cells for reprogramming, even a rough statistical estimate of efficiency would be helpful. Figure 7 states that 50k cells were plated for reprogramming. How many AP+ colonies were derived per experiment? How did the number of AP+ colonies vary between both biological replicates? If the aim is to provide a platform which others may use to derive stable pluripotent cell lines from blood-derived fibrocytes, I think this informations will be key to indicate the extend of time and money involved in this work.

Response: We appreciate the reviewer's suggestion. However, we respectfully believe that formal comparisons of reprogramming efficiency fall outside the scope of the present study. The objective of Figure 7 is to establish proof-of-principle that blood-derived fibrocytes can be reprogrammed toward induced pluripotency, rather than to optimize or benchmark reprogramming efficiency.

A rigorous assessment of reprogramming efficiency would require a dedicated experimental design involving multiple donor animals, standardized reprogramming conditions, and sufficient biological replicates to account for the well-recognized variability in colony formation between donors and experiments. Such studies would also require predefined criteria for colony scoring and statistical analysis. These experiments are substantially beyond the scope of the current manuscript, which is focused on establishing fibrocytes as a distinct blood-derived somatic cell platform.

Nevertheless, our experience indicates that fibrocytes generate AP-positive colonies without any problems similar to fibroblasts. This qualitative observation is consistent with our conclusion that fibrocytes represent a practical source of cells for reprogramming, although we have intentionally avoided making quantitative claims that are not supported by a study specifically designed for efficiency measurements. We have therefore retained the proof-of-principle presentation of these data.

Comment: The authors also highlight the complexity of choosing "one fibroblast control". I appreciate their reasoning. In light of this complexity, I would encourage the authors to provide

© 2026. Published by The Company of Biologists under the terms of the Creative Commons Attribution License (https://creativecommons.org/licenses/by/4.0/).

explicit justification for their choice of a fibroblast RNAseq dataset used for transcriptional comparision in figure 6, which is currently missing.

I raised questions regarding the documentation and explanation of the work presented in figure 6. The authors state they provided clarification in the figure legend and methods. I can only identify a minor change in the first sentence of the figure legend and would ask the authors to point out additional changes which I am missing.

Response: We appreciate the reviewer returning to this point. The purpose of Figure 6 is not to define a universal fibroblast transcriptional profile, but rather to provide a representative benchmark against which the distinct transcriptional identity of fibrocytes can be visualized. Because fibroblasts are themselves heterogeneous across tissues and physiological contexts, no single fibroblast population can serve as a universal reference. The dataset used here was selected because it represents one of the higher-quality, well-characterized publicly available bovine fibroblast RNA-seq datasets and therefore provides a robust and transparent reference for this comparison. We recognize that our previous revisions may not have made these clarifications sufficiently explicit. We have therefore revised the Methods to more clearly describe the comparator dataset.

Comment: The authors also highlight that fibrocyte markers can vary: "Fibrocyte marker expression varies substantially across species, isolation methods, culture conditions, activation states, and analytical platforms."

Under these circumstances especially I would encourage the authors to identify which genes are found in mouse and human fibrocytes, as well as in bovine. I agree with the authors to not overstate these as a fixed marker set, but they might still guide the comparison and grow the field.

Response: We agree that a cross-species comparison can provide useful context, provided that these genes are not interpreted as a fixed fibrocyte marker set. We have therefore added a comparison of fibrocyte-associated genes reported in human and mouse with those identified in bovine fibrocytes. Across species, fibrocytes share a conserved combination of hematopoietic/myeloid features (e.g., CD45/PTPRC and CD11b/ITGAM), extracellular-matrix programs (including COL1A1, COL3A1 and FN1), and chemokine/migratory signalling, although individual markers such as CD34, CD45 and CXCR4 vary substantially with differentiation state and culture conditions. The bovine transcriptome recapitulates this broader fibrocyte architecture, while also identifying species- and state-specific features. We have incorporated this comparison into the Discussion to provide a cross-species framework without implying a universal fibrocyte marker signature.

Comment: The authors state that the components of FcB medium were chosen for known roles in various cell biological aspects. I would be in favour of including this reasoning into the manuscript, preferrably with specific justifications for the signalling pathways and molecules chosen.

Response: We appreciate the reviewer's suggestion. The components of FbC medium were selected based on their established roles in regulating key cellular processes relevant to fibrocyte derivation and maintenance, including proliferation, extracellular matrix remodeling, TGF-β signaling, and cell plasticity. We agree that this rationale provides useful context and have now incorporated a brief justification for each component in the Methods section.

Reviewer 2

Comment: I appreciate the authors' detailed response and agree that the experiments done in the study supports the presence of a fibrocyte-like population. I also recognize the challenges associated with defining bovine fibrocytes and the current limitations in established bovine-specific protein markers. That said, my main concern was not the pathway analysis itself, but rather that the evidence for cell identity remains largely transcriptomic and indirect. Detection of hematopoietic and mesenchymal transcripts by RNA-seq, even when combined with morphology and culture characteristics, does not fully replace orthogonal protein-level validation, particularly when proposing a novel cell identity in a new species context.

© 2026. Published by The Company of Biologists under the terms of the Creative Commons Attribution License (https://creativecommons.org/licenses/by/4.0/).

Given the scope of the study and the authors' clarification, I do not consider additional experiments strictly necessary for publication. However, I recommend that the language throughout the manuscript be moderated where appropriate, and by acknowledging in the Discussion that definitive protein-level validation of bovine fibrocyte identity remains to be established.

Response: We respectfully disagree with reducing the derived cells to "fibrocyte-like" throughout the manuscript. The manuscript does not rely solely on transcriptomic inference. It establishes fibrocyte derivation using convergent evidence from blood origin, culture behavior, morphology, expansion, time- lapse emergence from adherent blood-derived colonies, transcriptomic separation from fibroblasts, immune/reparative pathway enrichment, upstream regulator analysis, receptor-level analysis, and uncovering fibrocyte-specific functional gene networks.

The manuscript then demonstrates that these cells are molecularly distinct from fibroblasts: "Samples from three independent animals clustered tightly within each group, indicating high within-group consistency and robust separation between fibrocyte and fibroblast transcriptomes." It further states that receptor and transcriptional networks distinguish "fibrocytes as a molecularly distinct somatic lineage rather than a fibroblast variant."

We acknowledge that protein-level validation can further refine bovine fibrocyte definitions in future studies, particularly because bovine-specific fibrocyte marker standards are limited. However, the absence of a universally accepted bovine fibrocyte protein marker panel does not negate the convergent evidence presented here. We have added a concise limitation to this effect without weakening the central conclusion that bovine peripheral blood-derived fibrocytes were derived and characterized.

Comment: I also appreciate the authors' clarification regarding the intended scope of the reprogramming experiments as proof-of-principle rather than comprehensive characterization of fully validated bovine iPSC lines. I agree that extensive pluripotency validation may be beyond the scope of the current study. However, the manuscript should consistently avoid language implying definitive establishment of fully characterized pluripotent stem cell lines unless additional protein-level or functional pluripotency assays are provided.

Response: We have reviewed the manuscript to ensure that language does not overstate the reprogramming experiment as a complete characterization of stable bovine iPSC lines. However, the evidence presented is sufficient for the manuscript's central claim that fibrocytes are reprogrammable toward induced pluripotency.

Comment: Thank you for the clarification regarding the scale bars. However, in Figure B the Day 7 panel appears to be an independent image rather than a magnified inset, and no scale bar is shown. Similarly, in Figure C only one of the two panels contains a scale bar. For clarity and consistency, please add scale bars to all independently displayed microscopy panels.

Response: We have added scale bars to all independently displayed microscopy panels, including the panels noted by the reviewer.

Third decision letter

MS ID#: bio.062486R2

MS Title: Signal-integrated control of immune and reparative programs establishes blood-derived fibrocytes reprogrammable toward induced pluripotency

Authors: Hannah Sylvester; Prasanthi P. Koganti; Shailesh Gurung; Viju V. Pillai; Vimal Selvaraj

© 2026. Published by The Company of Biologists under the terms of the Creative Commons Attribution License (https://creativecommons.org/licenses/by/4.0/).

I am happy to tell you that your manuscript has been accepted for publication in Biology Open, pending our standard publication integrity checks. It was accepted on 25th August 2026.

© 2026. Published by The Company of Biologists under the terms of the Creative Commons Attribution License (https://creativecommons.org/licenses/by/4.0/).
